# The role of eplet matching in solid organ transplantation

**DOI:** 10.3389/frtra.2025.1710058

**Published:** 2025-12-08

**Authors:** Viola A. Stögner, Dean M. Pucciarelli, Lauren Harkins, Adam Littleton, Richard Formica, Bohdan Pomahac, Siba Haykal

**Affiliations:** 1 Division of Plastic & Reconstructive Surgery, Department of Surgery, Yale School of Medicine, New Haven, CT, United States; 2 Department of Plastic, Aesthetic, Hand and Reconstructive Surgery, Burn Center, Hannover Medical School, Hannover, Germany; 3 Rutgers, Robert Wood Johnson Medical School, New Brunswick, NJ, United States; 4 Department of Biomedical Engineering, Yale University, New Haven, CT, United States; 5 Yale School of Medicine, New Haven, CT, United States; 6 Department of Internal Medicine, Section of Nephrology, Yale School of Medicine, New Haven, CT, United States

**Keywords:** eplet matching, molecular HLA matching, donor-recipient compatibility, alloimmune risk stratification, solid organ transplantation

## Abstract

**Introduction:**

Donor–recipient compatibility remains a central determinant of transplant success, yet conventional antigen-level human leukocyte antigen (HLA) matching provides limited resolution for predicting alloimmune risk. Molecular matching at the eplet level, which quantifies structural motifs on HLA molecules recognized by B- and T-cells, has emerged as a promising strategy to refine immunologic risk assessment.

**Methods:**

We conducted a scoping review of 98 studies encompassing 286,101 solid organ transplant (SOT) recipients across kidney, heart, lung, liver, pancreas, and combined grafts. Data on HLA typing approaches, eplet mismatch (epMM) algorithms, thresholds, and associations with clinical outcomes were systematically extracted and synthesized.

**Results:**

The majority of studies were retrospective kidney transplant cohorts, though evidence from heart, lung, and liver transplantation is expanding. Across organs, higher class II epMM burden—particularly at HLA-DQ and HLA-DR—was consistently associated with *de novo* donor-specific antibodies, antibody mediated rejection, and graft dysfunction. Reported epMM thresholds varied but were most robust for class II loci, while findings for class I loci were less consistent. Observed differences in epMM thresholds and effect sizes reflected both organ-specific immunobiology and methodological heterogeneity, including variation in typing resolution, mismatch algorithms, immunosuppression exposure, and study design.

**Conclusion:**

Eplet matching demonstrates significant potential to improve risk stratification and long-term graft outcomes across SOT. However, clinical translation is limited by inconsistent methods, equity concerns, and the absence of standardized epMM thresholds. Prospective studies, harmonized molecular typing, and integration with allocation frameworks are needed to establish the clinical utility and policy implications of molecular-level HLA matching.

## 1 Introduction

Human leukocyte antigen (HLA) compatibility remains a cornerstone of donor–recipient selection in transplantation. In routine clinical practice, HLA antigen typing, supported by antibody screening and final crossmatching, currently still represents the operational standard. This approach prioritizes speed and safety in the process of organ allocation, which are particularly relevant for deceased donors, while ensuring that preformed donor-specific antibodies (DSAs) are avoided. However, the low-resolution of HLA antigen typing has several limitations in predicting donor-recipient immunologic incompatibilities and their sequelae—raising discussions about required changes in transplant allocation policies for safer risk stratification (1, 2).

Eplet matching, along with high-resolution typing techniques, attempts to address this gap by quantifying structural motifs on HLA molecules that constitute B-cell–accessible epitopes. Eplets are small clusters of polymorphic amino acids on the solvent-exposed surface of an HLA protein; disparity at these motifs is hypothesized to drive alloantibody formation through direct B-cell recognition and T-cell help (3). The HLAMatchmaker algorithm formalized this concept by using high-resolution HLA allele sequences to detect and compare eplet mismatches (epMMs) between donor and recipient, while using a registry-based approach (https://epregistry.com.br) to further enumerate antibody-verified (immunologically relevant) eplets (2, 4). While the HLAMatchmaker is the most widely used approach for eplet matching, multiple complementary algorithms exist that operationalize molecular matching. The Predicted Indirectly ReCognizable HLA Epitopes (PIRCHE) algorithm models the T-cell–dependent, indirect pathway of allorecognition, predicting donor-derived HLA peptides presented by recipient HLA class II. The combined use of the PIRCHE-derived T-cell epitope mismatch score and HLAMatchmaker-derived epMM score has been promoted by several studies (5–7).

Emerging data and evidence, predominantly in the field of kidney transplantation but also in other types of organ transplantation, indicate associations of higher epMM burdens with *de novo* DSA (dnDSA) development, rejection, and impaired long-term graft performance and survival (2, 8). In particular, observational data is linking class II eplet or epitope mismatches with dnDSA, antibody mediated rejection (AMR), transplant glomerulopathy, and graft loss, while analogous associations are increasingly reported in heart and lung cohorts (9). In contrast, evidence in areas such as liver transplantation remain mixed. Notably, several transplant centers have piloted or integrated eplet-informed risk stratification and in some settings eplet-aware donor selection (e.g., University of Pittsburgh Medical Center, Seattle Children's, University of Minnesota) (10–13). However, implementation remains heterogeneous, with methodological variation (typing resolution, algorithm/version, epitope curation), organ-specific immunobiology, equity concerns related to HLA diversity, and uncertain operational thresholds posing challenges for clinical practice and policy. Recent analyses also underscore that moving from antigen- to allele-level matching alone does not guarantee better prediction, reinforcing the rationale for epitope-focused approaches (14–16).

This scoping review will map and critically appraise the extent, nature, and quality of evidence on eplet matching across solid organ transplantation (SOT), delineating reported organ- and loci-specific clinical outcomes and epMM thresholds, as well as methodological differences and existing gaps that must be addressed to translate eplet matching into general, outcome-oriented clinical practice.

## 2 Methods

### 2.1 Search protocol

A systematic search query was entered into the PubMed database on July 29th, 2025, resulting in a total of 383 articles. All articles were imported into Covidence for preliminary title, abstract and full-text screening. Following exclusions (see below criteria), 97 studies underwent data extraction and subsequent analyses.

### 2.2 Inclusion criteria

All prospective and retrospective clinical studies examining eplet matching, by use of the HLAMatchmaker algorithm, in the context of SOT, were included in the analysis. Articles that had utilized both the HLAMatchmaker as well as additional HLA-matching algorithms were included in the study.

### 2.3 Exclusion criteria

A total of 286 articles were excluded. Of these, 244 exclusions were due to articles not meeting inclusion criteria. The remaining 42 articles were excluded for any of the following reasons: study of non-organ cellular transplants (predominantly hematopoietic stem cell transplantation), studies that combined multiple types of transplantation without distinguishing results by individual graft-type, different publications reporting similar outcomes on the same patient cohorts (same authors with identical recipient demographics), or studies of non-vascular composite allograft transplantation.

### 2.4 Analysis

Of the 97 articles included for analyses, descriptive and HLA or epMM associated variables were compiled for a total of 284,540 organ transplant recipients. All data compilation and analyses were completed with assistance of Microsoft Excel with ToolPak. Average article-reported total, HLA class I, and class II epMMs were weighed by fraction of study participants for each study within the entire associated cohort. Analysis included study-related demographics, methodological differences, post-transplantation outcomes, in addition to relationships between outcomes, HLA-typing or eplet matching methods, individual eplet loci, epMM numbers, and mismatch thresholds.

## 3 Results

### 3.1 General results and demographics

After abstract and full-text screening a total of 97 articles, comprising 284,540 transplant recipients, were included in this review (Supplementary Table S1). Most articles involved retrospective studies (93.8%), while cohort studies (81.4%) represented the predominant study design, followed by registry-based studies (10.3%), case studies (7.2%), and case-control studies (1%). Five (5.2%) prospective studies were included. With 74.3% among reported articles, the kidney was the most frequent organ of transplantation, besides some reported data on heart (8.2%), lung (8.2%), and liver (7.2%) transplantation, and only few articles on pancreas transplantation (2.1%). The predominant study populations reported were male (60.8%) and adult (48.3%) organ recipients. Most of the pediatric studies involved heart transplantation (88%), followed by kidney (10%), and liver transplantation (3%). The mean overall recipient age was 41.3 years ± 15.4 (range 1.4–63), while mean age among adult recipients amounted 46.9 years ± 8 (range 18–63) and 8.7 years ± 4.3 (range 1.4–14) among pediatric recipients. For donors the mean overall reported age was 44.2 years ± 11.3 (range 6–58.5), and 46.7 years ± 8.2 (range 22.6–58.5) and 24.2 years ± 19.8 (range 6–42.5) for adult and pediatric donors respectively. Studies included both living and deceased donors, as well as primary transplantations and retransplantations. The mean follow-up period included 4.6 years ± 3 (range 0.1–15), with the longest follow up periods reported for lung (mean follow up 5.5 years) and the shortest for pancreas transplantations (mean follow up 3.3 years). Reported immunosuppressive regimens, included triple therapy in 49.5% of articles, monotherapy in 6.2%, dual therapy in 4.1% and steroid-free therapy in 1% of articles, while 39.2% of studies described varying regimens or did not specify immunosuppressive treatments (Table 1).

**Table 1:** Descriptive data on all included studies in primary scoping analysis stratified by organ or tissue graft.

Outcome parameter	Descriptive statistics, by Graft					
	Kidney	Heart	Pancreas	Lung	Liver	Total
General (n, studies, or patients*, %)						
*n*—studies (total)	72	8	2	8	7	97
Retrospective (*n*—studies, %)	68 (94.4%)	8 (100%)	2 (100%)	8 (100%)	6 (85.7%)	91 (93.8%)
Prospective (*n*—studies, %)	4 (5.6%)	0(%)	0 (0%)	0 (0%)	1 (14.3%)	5 (5.2%)
Case studies (*n*—studies, %)	7 (9.7%)	0 (0%)	0 (0%)	0 (0%)	0 (0%)	7 (7.2%)
Cohort study (*n*—studies, %)	58 (80.6%)	5 (62.5%)	2 (100%)	8 (100%)	7 (100%)	79 (81.4%)
Registry-based (*n*—studies, %)	6 (8.3%)	3 (37.5%)	0 (0%)	0 (0%)	0 (0%)	10 (10.3%)
Case-control (*n*—studies, %)	1 (1.4%)	0 (0%)	0 (0%)	0 (0%)	0 (0%)	1 (1%)
*n*—recipients (total)*	237,700	43,749	86	1,313	1,692	284,540
Male	139,742 (58.8%)	31,610 (72.3%)	43 (50%)	764 (58.2%)	764 (64.1%)	172,905 (60.8%)
Adult*	98,842 (41.6%)	36,112 (82.5%)	86 (100%)	1,264 (96.3%)	938 (55.4%)	137,242 (48.3%)
Pediatric*	764 (0.3%)	7,089 (16.2%)	0 (0%)	0 (0%)	214 (12.6%)	8,067 (2.8%)
Mixed age cohort or unspecified*	138,094 (58.1%)	548 (1.3%)	0 (0%)	49 (3.7%)	540 (32%)	139,231 (48.9%)
Recipient average age (years);^$^	41.4 (Range: 9–63, *SD* *=* *13.5*)	32 (Range: 4.3–53.8, *SD* *=* *25.1*)	46 (Range: N/A, *SD* N/A)	52.4 (Range: 48–55.73, *SD* *=* *2.5*)	33.4 (Range: 1.4–55, *SD* *=* *27.3*)	41.3 (Range: 1.4–63, *SD* *=* 15.4)
Adult*	45.3 (Range: 18–63, *SD* *=* *8.5*)	50.3 (Range: 46–53.8, *SD* *=* *4*)	46 (Range: N/A, *SD* N/A)	52.4 (Range: 48–55.73, *SD* *=* *2.5*)	3.6 (Range: 50.94–55, *SD* *=* *3.1*)	46.9 (Range: 18–63, *SD* *=* 8)
Pediatric*	13.5 (Range: 9–14, *SD* *=* *11.7*)	4.7 (Range: 4.3–5, *SD* *=* *0.5*)	–	–	53.3 (Range: 1.4–5.8, *SD* *=* *2.1*)	8.7 (Range: 1.4–14, *SD* *=* 4.3)
Intra-study mixed age cohort, or unspecified*	45.5 (Range: N/A, *SD* *=* *6.8*)	–	–	–	–	45.5 (Range:39—51.9, SD *=* 6.8)
Donor average age (years);^$^	46.9 (Range: 22.6–58.5, *SD* *=* *7.5*)	23 (Range: 6–35, *SD* *=* *14.6*)	46.3 (Range: N/A, *SD* N/A)	45 (Range: None/same, *SD* *=* *0*)	51.25 (Range: 44–58.5, *SD* *=* *10.3*)	44.2 (Range: 6–58.5, *SD* *=* 11.3)
Adult*	47.9 (Range: 22.6–58.5, *SD* *=* *7.7*)	33.6 (Range: 30.8–35, *SD* *=* *2.4*)	–	45 (Range: None/same, *SD* *=* *0*)	51.3 (Range:44–58.5, *SD* *=* *10.3*)	46.7 (Range: 22.6–58.5, *SD* = 8.2)
Pediatric*	41.3 (Range: 40–42.5, *SD* *=* *1.8*)	7.1 (Range: 6–8.2, *SD* *=* *1.6*)	–	–	–	24.2 (Range: 6–42.5, *SD* *=* 19.8)
Mixed age cohort or unspecified*	42.8 (Range: 39.2–48, *SD* *=* *4.2*)	–	–	–	–	42.8 (Range: 39.2–48, *SD* *=* 4.2)
Follow-up (avg, years)	4.6 (Range: 0.1–15, *SD* *=* *3*)	5.4 (Range: 2.2–10, *SD* *=* *3*)	3.3 (Range: N/A, *SD* NA)	5.5 (Range: 1–7.8, *SD* *=* *3.9*)	3.7 (Range: 1.7–6.8, *SD* *=* *2.8*)	4.6 (Range: 0.1–15, *SD* *=* 3)
*Donor type (n—studies, %)*						
Living	11 (15.3%)	0 (0%)	–	0 (0%)	0 (0%)	11 (11.3%)
Deceased	12 (16.7%)	8 (100%)	–	6 (75%)	2 (28.6%)	28 (28.9%)
Mixed in cohort	38 (52.7%)	0 (0%)	–	0 (0%)	4 (57.1%)	42 (43.3%)
Unspecified	11 (15.3%)	0 (0%)	2 (100%)	2 (25%)	1 (14.3%)	16 (16.5%)
*Transplantation type (n—studies, %)*						
Primary	27 (37.5%)	6 (75%)	0 (0%)	2 (25%)	5 (71.4%)	40 (41.3%)
Re-transplantation	4 (5.6%)	0 (0%)	0 (0%)	0 (0%)	0 (0%)	4 (4.1%)
Mixed in cohort	25 (34.7)	0 (0%)	1 (50%)	0 (0%)	1 (14.3%)	27 (27.8%)
Unspecified	16 (22.2%)	2 (25%)	1 (50%)	6 (75%)	1 (14.3%)	26 (26.8%)
*Immunosuppressive Regimen (n—studies, %)*						
Monotherapy	3 (4.2%)	1 (12.5%)	0 (0%)	0 (0%)	2 (28.6%)	6 (6.2%)
Dual therapy	4 (5.6%)	0 (0%)	1 (50%)	0 (0%)	0 (0%)	4 (4.1%)
Triple therapy	38 (52.7%)	2 (25%)	0 (0%)	7 (87.5%)	2 (28.6%)	48 (49.5%)
Steroid-free therapy	0 (0%)	0 (0%)	1 (50%)	0 (0%)	0 (0%)	1 (1%)
Induction therapy (reported)	43 (59.7%)	2 (25%)	2 (100%)	4 (50%)	5 (71.4%)	56 (57.7%)
Varied in cohort, or unspecified	27 (37.5%)	5 (62.5%)	0 (0%)	1 (12.5%)	3 (41.8%)	38 (39.2%)
*Eplet Mismatches* ^#^						
Total, weighted avg (*n*; range, SD)	24.7 (5,219; Range: 3.5–41, *SD* *=* *13.1*)	27 (77; Range: N/A, *SD* *=* *N/A*)	–	42.8 (759; Range: 8.3–36.8, *SD* *=* *26.2*)	–	27 (6,055; Range: 3.5–41, *SD* *=* *17.4*)
Class I, weighted avg (*n*; range, SD)	11.8 (2,553; Range: 4–49, *SD* *=* *10*)	14.6 (6,095; Range: 8–10, *SD* *=* *4.3*)	–	13.7 (517; Range: 11–16.8, *SD* *=* *4.1*)	12.9 (122; Range: 12.8–13, *SD* *=* *0.15*)	13.8 (9,287; Range: 4–49, *SD* *=* *8.8*)
Class II, weighted avg (*n*; range, SD)	10.9 (4,925; Range: 1–23.3, *SD* *=* *7.5*)	11.4 (6,018; Range: 5–12.9, *SD* *=* *5.6*)	–	20.5 (700; Range: 14.5–25, *SD* *=* *5.3*)	39 (79; Range: N/A, *SD* *=* *N/A*)	11.9 (11,722; Range: 1–39, *SD* *=* *8.8*)
*HLA Typing Methods, by Graft (n—studies, %)*						
	**Kidney**	**Heart**	**Pancreas**	**Lung**	**Liver**	**Total (*n* = 97)**
SSO only	20 (27.8%)	3 (37.5%)	1 (50%)	1 (12.5%)	3 (42.8%)	28 (28.9%)
SSP only	4 (5.6%)	0 (0%)	0 (0%)	1 (12.5%)	1 (14.3%)	6 (6.2%)
SSOP only	5 (6.9%)	0 (0%)	0 (0%)	0 (0%)	0 (0%)	5 (5.2%)
SBT only	0 (0%)	0 (0%)	0 (0%)	2 (37.5%)	0 (0%)	2 (2.1%)
OP only	1 (1.4%)	0 (0%)	0 (0%)	0 (0%)	0 (0%)	1 (1%)
NGS only	8 (11.1%)	1 (12.5%)	0 (0%)	2 (37.5%)	1 (14.3%)	12 (12.3%)
Serological only	3 (4.2%)	0 (0%)	0 (0%)	0 (0%)	0 (0%)	3 (3.1%)
>1 employed	16 (22.2%); 6 including NGS	0 (0%)	1 (50%); 0 including NGS	2 (25%); 0 including NGS	1 (14.3%); 0 including NGS	20 (20.6%); 6 including NGS
Unspecified	15 (20.8%)	4 (50%)	0 (0%)	0 (0%)	1 (14.3%)	20 (20.6%)
*Use of Matching Algorithms in Addition to HLA Matchmaker*						
	**Kidney**	**Heart**	**Pancreas**	**Lung**	**Liver**	**Total (*n* = 97)**
PIRCHE	12 (17.7%)	1 (12.5%)	1 (50%)	2 (25%)	2 (28.6%)	18 (18.6%)
HLA-EMMA	5 (6.9%)	1 (12.5%)	0 (0%)	1 (12.5%)	0 (0%)	7 (7.2%)
SNOW	1 (1.4%)	0 (0%)	0 (0%)	0 (0%)	0 (0%)	1 (1%)
TCE algorithm	1 (1.4%)	0 (0%)	0 (0%)	0 (0%)	0 (0%)	1 (1%)

* *n* refers to number of patients as opposed to studies.

# Data weighed by individual study participants (*n*).

$ Due to available data, standard deviations are of average age across several studies, not average age of individual transplant recipients.

Σ [(individual study *n*/total analysis *n*) × (avg category-respective mismatches)].

Non-whole transplantation was included within respective categories (i.e., pancreatic islet transplant within pancreatic transplant strata).

SD, standard deviation; SSO, single strand oligonucleotide assay; SSP, sequence specific primers; SSOP, sequence specific oligonucleotide probe; OP, oligonucleotide probe; NGS, next generation sequencing; SBT, sequence-based typing; PIRCHE, Predicted Indirectly ReCognizable HLA Epitopes; HLA-EMMA, HLA Epitope Mismatch Algorithm; SNOW, Snowflake; TCE, T-cell epitope.

### 3.2 Eplet matching

The reported HLAMatchmaker results amounted for an overall mean epMM count of 27 ± 17.4 (range 3.5–41), and 13.8 ± 8.8 (range 4–49) and 11.9 ± 8.8 (range 1–39) for HLA class I and class II molecules respectively. Mean epMM counts in kidney transplantation specifically were 24.7 ± 13.1 (range 3.5–41) total, 11.8 ± 10 (range 4–49) for class I, and 10.9 ± 7.5 (range 1–23.3) for class II. About 16% of studies utilized exclusively high-resolution next generation sequencing (NGS) (12.3%) or sequence-based typing (SBT) (2.1%), while the rest employed intermediate to low-resolution HLA typing methods, including single strand oligonucleotide assay (28.9%), sequence specific primers (6.2%), sequence specific oligonucleotide probe (5.2%), HLA serology (3.1%), oligonucleotide probe (1%), or used more than one method (20.6%), or didn't specify the typing method (20.6%). Among the studies that used more than one HLA typing method, 30% included NGS (Table 1).

While all articles employed the HLAMatchmaker for eplet matching, some articles used additional HLA matching algorithms. Specifically, 18 studies (18.6%) included the PIRCHE, 7 (7.2%) the HLA Epitope Mismatch Algorithm (HLA-EMMA), and 1 study (1%) employed the Snowflake algorithm (SNOW) or T-cell epitope (TCE) algorithm respectively for additional HLA matching results (Table 1).

### 3.3 Eplet mismatches & adverse transplant outcomes

93% of included articles analyzed HLA class II and 86% HLA class I epMMs in the context of transplant outcomes. Among those articles 70% reported significant associations between HLA class II epMMs and transplant outcomes, while only 24.1% found significant relationships with HLA class I epMMs. Analysis of specific HLA loci revealed that HLA-DQ epMMs were most frequently significantly associated with adverse transplant outcomes (61.8%), followed by HLA-DR (38.8%) (Table 2).

**Table 2:** Incidence of eplet loci (fraction of total articles examining a particular eplet loci) associated with outcomes following transplantation, stratified by graft tissue type.

Incidence of eplet loci associated with transplant outcomes (% of all respective studies)								
Graft type	HLA class I (total)	A	B	C	HLA Class II (total)	DR	DQ	DP
Kidney	13/61 (21.3%)	8/59 (13.6%)	8/59 (13.6%)	4/38 (10.5%)	46/67 (68.7%)	24/57 (42.1%)	34/56 (60.7%)	4/16 (25%)
Heart	3/8 (37.5%)	2/8 (25%)	2/8 (25%)	0/5 (0%)	7/8 (87.5%)	4/8 (50%)	4/5 (80%)	1/2 (50%)
Pancreas	0/1 (0%)	0/1 (0%)	0/1 (0%)	–	0/1 (0%)	0/1 (0%)	0/1 (0%)	–
Lung	1/8 (12.5%)	0/8 (0%)	0/8 (0%)	1/7 (14.3%)	6/8 (75%)	2/8 (25%)	6/8 (75%)	0/4 (0%)
Liver	3/5 (60%)	1/5 (20%)	1/5 (20%)	2/5 (40%)	4/6 (66.7%)	1/6 (14.3%)	3/6 (50%)	1/2 (50%)
Total	20/83 (24.1%)	11/81 (13.6%)	11/81 (13.6%)	7/55 (12.7%)	63/90 (70%)	31/80(38.8%)	47/76 (61.8%)	6/24 (25%)

Percentages represent total percent of studies with a found association with a particular HLA locus among all articles that had analyzed that locus. Thus, percentages do not add to a cumulative of 100%.

0 (0%), not associated; –, data not available.

The overall most common reported study endpoint associated with epMMs among transplant recipients was graft loss (*n* = 33,109), followed by T-cell mediated rejection (TCMR) (*n* = 7,861), graft dysfunction (*n* = 3,046), dnDSA (*n* = 2,646), AMR (*n* = 1,676), and CD4 positive staining (*n* = 64). When sorting study endpoints by incidence, however, the order was slightly different, with the highest incidence described for graft loss with 27%, followed by CD4 positive staining (20%), TCMR (19%), dnDSAs (15%), AMR (11%), and graft dysfunction (2%). When looking at significant results only, dnDSA development, found in 35 articles, was the most frequently reported study endpoint associated with epMMs, followed by TCMR (16 articles) and AMR (14 articles) (Table 3).

**Table 3:** Incidence of post-transplantation outcomes by graft tissue type, characterized by fraction and percent.

Incidence of outcomes, fraction of total						
Graft type	dnDSA	AMR	TCMR	Graft dysfunction	Graft loss	CD4 (+) staining
Kidney	2,033/14,881 (13.7%)*	1,413/12,211 (11.6%)*	1,514/6,389 (23.7%)*	801/121,466 (0.7%)*	30,926/86,479 (35.8%)*	64/315 (20.3%)
Heart	193/1,189 (16.2%)*	172/1,463 (11.8%)*	5,980/34,758 (17.2%)*	1,793/3,131 (69.3%)	2,171/37,812 (5.7%)*	–
Pancreas	28/86 (32.6%)*	–	–	–	–	–
Lung	118/443 (26.6%)*	54/190 (28.4%)*	113/285 (39.6%)*	405/853 (47.5%)	–	–
Liver	274/814 (33.7%)*	29/816 (3.6%)*	254/1,022 (24.9%)*	47/184 (25.5%)*	12/80 (15%)*	–
Total	2,646/17,413 (15.2%)*	1,676/14,680 (11.4%)*	7,861/42,454 (18.5%)*	3,046/125,634 (2.4%)*	33,109/124,371 (26.6%)*	64/315 (20.3%)
TAS epMM	35	14	16	7	7	0

* Statistically significant association between respective post-transplantation outcome and with epMM thresholds in ≥1 study.

TAS epMM, total articles with significant associations between outcome variable and epMM; dnDSA, *de novo* donor specific antibodies; AMR, antibody mediated rejection; TCMR, T-cell mediated rejection.

### 3.4 Significant epMM thresholds

#### 3.4.1 Liver

Only three articles report epMM thresholds in liver transplantation, of which two studies looked at the significance of epMM counts on dnDSA formation in the pediatric transplant cohorts. Shin et al. determined cutoffs of ≥20 HLA-DR or ≥22 HLA-DQ epMMs for the formation of HLA-DR and HLA-DQ dnDSAs respectively. In addition, they found that a combined threshold of ≥21 HLA-DR and HLA-DQ epMMs was also significantly related with dnDSA formation. Ekong et al. significantly associated a cutoff of >5 or >6 HLA-DQ epMMs with dnDSA formation and graft dysfunction. The third article by Guiral et al. identified a risk threshold of ≥2 HLA-C epMMs to be significant for TCMR.

#### 3.4.2 Lung

Only three studies report epMM thresholds in lung transplantation. Kleid et al. (2023) (17) found cutoffs of >42.5 epMMs (HLA-A, HLA-B, HLA-C, HLA-DRB1, HLA-DR345, HLA-DQB1, HLA-DPA1, HLA-DPB1) or >30.5 immunogenic epMMs or >85.50 PIRCHE-II 5 loci score or >560.00 PIRCHE-II 11 loci score as individual predictors of class II dnDSA formation. Hiho et al. (18) determined a combined risk threshold of >29 for the total epMM counts of HLA class I and II, as well as a threshold of >19 for class II epMMs only for both graft dysfunction and recipient survival. Conversely, Hirama et al. found a cutoff of 61 epMMs (HLA-A, HLA-B, HLA-C, HLA-DRB1, HLA-DQB1) to not be significant for patient survival, primary graft dysfunction, or acute rejection.

#### 3.4.3 Heart

Five studies report epMM thresholds for heart transplantation. Cardoso et al. identified a cutoff of >2 HLA-DPB epMMs as a predictor of graft dysfunction. Sullivan et al. reported a low risk <10, intermediate risk 10–20, and high risk >20 threshold for class I epMMs predicting graft dysfunction. Albers et al. determined a similar risk threshold of >20 class I epMMs for graft loss.

As a predictor of dnDSA formation, Mangiola et al. report individual thresholds of >13 class I epMMs or >9 HLA-DR epMMs or >6 HLA-DQ epMMs while Zhang X. et al. (19) created groups above and below a median HLA-DQ epMM of 18, revealing an insignificant trend (*p* *=* *.066).* Mangiola et al. also found a significant association of individual PIRCHE-II mismatch scores of 141 for class I, 116 for HLA-DR, and 111 for HLA-DQ with the development of dnDSAs.

AMR was associated with epMM counts of 9 for class I or 8 for HLA-DR or 8 for HLA-DQ by Mangiola et al. and with epMM counts of >21 for HLA-DQ by Zhang X. et al. (19). Albers et al. report a low risk <10, intermediate risk 10–20, and high risk >20 class II epMM threshold for AMR. Albers et al. further identified a class I epMM count of >20 as a predictor of rejection.

#### 3.4.3 Kidney

In kidney transplantation, 33 studies report threshold values for various clinical outcomes. Thresholds were based on differing epMMs (overall, Class I, Class II, HLA-A, HLA-B, HLA-DR, HLA-DQ, immunogenic epMMs) and PIRCHE-II scores. The most common reported significant thresholds included HLA-DQ, HLA-DR, and total epMMs, while five articles report a combination thresholding approach, including mismatch counts of at least two of the above epMM classes or PIRCHE-II scores. All thresholds reported in these studies can be found in Tables 4, 5.

**Table 4:** All reported epMM thresholds for clinical outcomes in kidney transplantation.

Number of specific EpMM thresholds and associated transplant outcomes										
Threshold parameter	dnDSA	AMR	TCMR	Graft loss/survival	Organ dysfunction	Deleterious effect	PRA	CXCL10	Rejection (unspecified)	Total number of thresholds per scoring method
Total/All epMM	6 (6*)	1 (0*)	1 (1*)	4 (2*)	1 (1*)	1 (1*)				14 (11*)
Class I	3 (1*)	1 (0*)	1 (0*)	3 (0*)					2 (2*)	8 (3*)
HLA-A	1 (0*)									1 (0*)
HLA-B	1 (1*)									1 (1*)
Class II	2 (2*)	1 (1*)	1 (0*)		1 (1*)					5 (4*)
HLA-DR	5 (3*)	3 (3*)	1 (0*)	2 (2*)	1 (1*)		1 (0*)		2 (2*)	13 (9*)
HLA-DQ	7 (7*)	4 (4*)	2 (0*)	1 (1*)	1 (1*)		1 (1*)	1 (1*)	2 (2*)	19 (17*)
Immunogenic epMM				1 (0*)						1 (0*)
PIRCHE-II	4 (4*)			2 (2*)						6 (6*)
Combination	5 (4*)	3 (1*)	3 (3*)	1 (1*)					1 (0*)	13 (9*)
Total number of thresholds per clinical outcome	34 (28*)	12 (9*)	8 (4*)	14 (8*)	4 (4*)	1 (1*)	2 (1*)	1 (1*)	7 (6*)	

* Number of thresholds (number of significant thresholds).

epMM, eplet mismatch, dnDSA, *de novo* donor specific antibody, AMR, antibody mediated rejection, TCMR, T-cell mediated rejection, PRA, panel reactive antibody.

**Table 5:** Eplet thresholds for clinical outcomes in kidney transplantation.

Specific epMM thresholds and associated transplant outcomes										
Eplets	dnDSA	AMR	TCMR	Graft loss/survival	Organ sysfunction	Deleterious effect	PRA	CXCL10	Rejection (unspecified)	epMM threshold range
Total/All epMM	>22 (20)* >12 (21)* >16 (22)* >5 (23)* >27 (24)* <5, ≥5–<18, ≥18–<36, ≥36 (25)*	low: 27–60, medium: 62–72, high: 73–129 (26)	>20 (27)*	0–6, 7–9, 10–12, ≥13 (28) >5 (23)* low: 27–60, medium: 62–72, high: 73–129 (26) <5, ≥5–<18, ≥18–<36, ≥36 (25)*	≥60 (29)*	>12 (21)*				5–73 5–60*
Class I	>8 (7)* >9 (30) 0–16, 17–29, >29 (31)	≥10 (32)	≥10 (32)	>70 (33) 0–16, 17–29, >29 (31) ≥10 (34)					>70 (33)* 0–16, 17–29, >29 (31)*	8–70*
HLA-A	≥8 (34)									
HLA-B	≥6 (34)*									
Class II	>3 (7)* >14 (35)*	low: ≤5, high: ≥13 (32)*	≥13 (32)		≥48 (29)*					3–48*
HLA-DR	>13 (36)* >5 (37)* >10 (38)* >3 (30) 0–10, 11–20, >20 (31)	≥6 (32)* >13 (36)* ≥15 (37)*	≥6 (32)	>10 (39)* 0–10, 11–20, >20 (31)*	>10 (9)*		>12 (40)		>10 (39)* 0–10, 11–20, >20 (31)*	3–20 5–20*
HLA-DQ	>9 (36)* ≥6 (41)* >8 (42)* >5 (37)* ≥11 (43)* >17 (38)* ≥2 (30)*	≥7 (32)* >9 (36)* ≥15 (37)* >3 (44)*	≥7 (32) >3 (44)	>17 (39)*	>17 (9)*		>12 (40)*	>3 (44)*	>17 (39)* low: 0, intermediate: 1–5, high: ≥6 (45)*	2–17*
Immunogenic epMM				0–5, 6–8, 9–11, ≥12 (28)						
PIRCHE-II	>41 (Class I), >30 (Class II) (7)* <9, ≥9–<35, ≥35–<90, ≥90 (25)* >176 (35)* >176 (23)*			>176 (23)* <9, ≥9–<35, ≥35–<90, ≥90 (25)*						9–176*
Combination	low: total epMM <73 + top 10 immunogenic epMM <4 +PIRCHEII <93; high: total epMM ≥73 + top 10 immunogenic epMM ≥4 +PIRCHEII ≥93 (46)^#^ low: DR <7 and DQ < 9, intermediate: DR ≥ 7 or DQ 9–14, high: DQ ≥15 (47)* low: DR <7 and DQ <9, intermediate: DQ < 15, high: DQ ≥ 15 (48)* low: DR = 0 and DQ = 0; intermediate: DR = 1–6 and/or DQ = 1–8, high: DR ≥ 7 or DQ ≥ 9 (49)*	low: total epMM <73 + top 10 immunogenic epMM <4 +PIRCHEII <93; high: total epMM ≥73 + top 10 immunogenic epMM ≥4 +PIRCHEII ≥93 (46) low: DR <7 and DQ < 9, intermediate: DR ≥ 7 or DQ 9–14, high: DQ ≥15 (47) low: DR = 0 and DQ = 0; intermediate: DR = 1–6 and/or DQ = 1–8, high: DR ≥ 7 or DQ ≥ 9 (49)*	low: total epMM <73 + top 10 immunogenic epMM <4 +PIRCHEII <93; high: total epMM ≥73 + top 10 immunogenic epMM ≥4 +PIRCHEII ≥93 (46)* low: DR 0–6 and DQ 0–8, intermediate: DR any, DQ ≤14, high: DR >14 (50)* low: DR = 0 and DQ = 0; intermediate: DR = 1–6 and/or DQ = 1–8, high: DR ≥ 7 or DQ ≥ 9 (49)*	low: DR = 0 and DQ = 0; intermediate: DR = 1–6 and/or DQ = 1–8, high: DR ≥ 7 or DQ ≥ 9 (49)*					low: DR <7 and DQ < 9, intermediate: DR ≥ 7 or DQ 9–14, high: DQ ≥15 (47)	

* Significant threshold.

# Significant threshold for dnDSA formation at 5 years but not significant at 1 year.

epMM, eplet mismatch, dnDSA, *de novo* donor specific antibody, AMR, antibody mediated rejection, TCMR, T-cell mediated rejection, PRA, panel reactive antibody.

### 3.5 *De novo* DSA formation

Significant thresholds based on total epMMs and predictive for dnDSA formation were reported in six articles, all of which were found to be statistically significant. However, these thresholds spread over a larger range, with the lowest counting >5 and the highest counting ≥36 epMMs. Seven papers identified HLA-DQ thresholds, ranging from ≥2 to >17 epMMs, as significant predictors for dnDSA formation. Four articles report thresholds for PIRCHE-II scores that were significantly associated with the presence of dnDSA. Of those four papers, two studies reported the same PIRCHE-II threshold of >176. Five articles reported HLA-DR cutoffs for dnDSA formation, but only three thresholds reached statistical significance (>5, >10, >13).

### 3.6 Rejection

Three studies identified HLA-DR epMM thresholds for AMR, all of which were significant (≥6, >13, ≥15). Four papers reported HLA-DQ epMM thresholds, all of which were significant (>3, ≥7, >9, ≥15). For TCMR, HLA class II epMM counts, HLA-DR epMM, and HLA-DQ epMM individually did not appear to be significant, but the combination of both HLA-DR and HLA-DQ epMMs revealed significance in predicting TCMR.

### 3.7 Graft loss/survival

Four articles investigated total epMM thresholds as predictor for graft loss/survival, while two of them report significant thresholds. In addition, three studies assessed class I epMM cutoffs in the context of graft loss/survival, but no thresholds were found to be significant.

Information about type of SOT, used matching algorithms, number of included transplant recipients and age groups, assess HLA loci, as well as found associations with transplant outcomes as well as identified thresholds are summarized by article in Supplementary Table S1.

## 4 Discussion

Solid organ transplantation has witnessed remarkable advances over the past decades, with improved surgical techniques, logistics, immunosuppressive regimens, and perioperative care leading to significantly better short-term outcomes and patient survival (51, 52). Yet despite these achievements, long-term graft survival has not improved at the same pace, and rejection—particularly AMR driven by DSAs—remains a major challenge. To date, donor-recipient compatibility, which plays a critical role in the process of dnDSA formation and associated poor long-term outcomes, is still primarily assessed through HLA antigen level typing combined with antibody screening and crossmatching. While these traditional matching strategies lack the molecular resolution to identify subtle HLA incompatibilities capable of triggering an alloimmune response, eplet matching of high-resolution HLA data has emerged as a promising approach towards more precise risk stratification and individualized donor-recipient allocation.

For this reason, eplet matching is being increasingly explored in clinical practice, particularly kidney transplantation, while some transplant centers have started incorporating epMM analysis in their matching processes, especially in the context of living donor transplants. In fact, the great majority of included articles in this review involved kidney transplantation, which, in contrast to heart and lung transplantation, is typically less urgent thanks to the efficacy of renal replacement therapy (53).

While eplet matching algorithms computationally achieve mismatch calculations very fast, relatively long turnaround times of high-resolution allele-level sequencing, in comparison to HLA antigen matching, represented a major obstacle to a broad clinical implementation of those technologies in SOT. The challenge of longer turnaround times appears especially critical in deceased donor transplantation. In contrast to SOT, high-resolution typing has established as the gold standard for donor selection in hematopoietic stem cell transplantation (HSCT) where even fine HLA allele differences can trigger graft-vs.-host disease and graft failure. Because HSCTs are almost always planned in advance, there is typically time to perform full donor and recipient high-resolution typing before the transplant (54). Recent technological advances are promising to overcome this bottleneck in clinically practical HLA typing. Novel methods like rapid NGS and point-of-care DNA typing, enable fast full-allele typing in only a few hours, holding the potential to replace low-resolution typing for deceased donor allocation in the future (55, 56). The emerging transformation of transplant practice towards routine high-resolution HLA typing open the doors to a broad use of eplet matching in organ allocation.

Several studies, the majority retrospective, have explored the predictive value of eplet matching on SOT outcomes, reporting specific epMM counts and thresholds amongst others—data that this review aims to contextualize for a possible translation into clinically applicable frameworks.

Overall, relevant associations with adverse transplant outcomes were most frequently found for HLA class II epMMs compared to HLA class I epMMs. Specifically, the DQ locus showed the most consistent association with study endpoints, particularly dnDSA development. Similarly, clinically relevant threshold values for epMMs were more commonly reported for HLA class II than class I. This discrepancy is likely in part reflective of the fact that more studies reported on HLA class II epMMs than class I (90). Described thresholds for HLA class II epMMs most frequently involved individual HLA loci (for example, HLA-DR or HLA-DQ) as opposed to overall cutoffs for class II epMMs. The inverse was true for class I epMM thresholds, which were commonly reported as overall cutoffs for all class I epMMs together.

In kidney transplantation, which accounts for most available data on epMMs in SOT, HLA-DQ involved the highest count of reported epMM thresholds, while 17 out of the 19 described cutoffs showed statistical significance. Reported thresholds for HLA-DQ ranged from ≥2 to ≥17, most of them relating to dnDSA development (*n* = 6), and AMR (*n* = 4), and all of them reaching statistical significance (Tables 4, 5). One article reported a significant relationship between HLA-DQ epMMs and urinary CXCL10 expression, a biomarker for rejection in kidney transplantation (44). HLA-DR was the second most common HLA locus associated with transplant outcomes. A total of 13 HLA-DR epMM thresholds have been described, nine of them statistically significant. Most common related transplant outcomes were dnDSA development with three out of five statistically significant thresholds, and AMR with three out of three statistically significant thresholds, ranging from >3 to >13 and ≥6 to ≥15 respectively. The presence of HLA class II dnDSAs has been critically linked to rejection, particularly AMR, and inferior long-term transplant outcomes (89). In fact, the greatest specificity for chronic AMR in kidney, liver, heart and lung transplantation has been associated with HLA-DQ antibodies, highlighting the relevance and potential of eplet matching in the organ allocation process (42, 57, 58). These findings align with described observations that HLA class I dnDSAs appear particularly in the earlier phase after transplantation, often changing to negative after the first posttransplant year, while HLA class II dnDSAs are generally more common and persistent.

Among articles reporting significant associations between epMMs and adverse outcomes following transplantation, the most reported (by number of articles) adverse outcomes were dnDSA development, TCMR, and AMR. However, graft loss had the highest incidence of adversity among reported individual transplant recipients in kidney transplantation, despite only being found significant in seven articles, potentially indicating the need for wider inclusion of this endpoint in future studies involving other types of SOT as well (Table 3). Within the kidney transplantation cohort, CD4 + histologic staining (typically in peritubular vasculature) had a similar incidence among transplant recipients as compared to TCMR; however, CD4 + staining was not significantly associated with epMM thresholds, while TCMR was. These findings suggest that CD4+ T-cell presence in graft tissue does not correlate with epMMs, whereas clinically defined T-cell mediated rejection does. Thus, epMM may reflect the risk of alloimmune activation rather than simple T-cell infiltration, underscoring the complex link between molecular mismatch and cellular immune responses (59, 60). The incidence of graft dysfunction in heart transplantation was distinctly higher than remaining outcome variables, which were otherwise more homogenous in incidence among transplant recipients (Table 3). Within the included studies lung and liver transplantation show a tendency towards higher incidences of dnDSA development and TCMR among recipients, as compared to kidney and heart transplantation (Table 3).

The total weighted average epMM count for HLA class I was 13.8 and 11.9 for HLA class II mismatches, while ranges of 4–49 and 1–39 respectively illustrate the variance across studies but also type of organ. In fact, higher weighted average HLA class II epMM counts have been reported for lung (20.5 epMMs) and liver transplant (39 epMMs). These observations, as well as variations in described epMM thresholds across organ types could be related to organ-specific immunological differences, while methodological variations among included studies must be factored in as possible cofounding variables as well. An immunogenic hierarchy between different organs has been suggested by Phillips and Callaghan (61), underlining a possible need for organ-specific epMM thresholds. While small bowel transplants, due to their lymphoid load, generally require the most aggressive immunosuppressive regimens, heart and lung appear more immunogenic than kidney and liver (61, 62). The liver, in fact, is unique in its tolerogenic milieu, maintained by its specialized endothelial and immune cells, as well as the secretion of soluble HLA, capable of eliminating and neutralizing antibodies, especially class I antibodies. Unlike the liver, the lung lacks these tolerogenic mechanism, while being under constant environmental and hence antigen exposure, which makes it a highly immunogenic organ in transplantation (57, 63).

While these biologic and immunologic distinctions between transplanted organs make differences in epMM cut offs likely, the current lack of data on this topic in any type of SOT other than kidney transplantation, unfortunately, does not allow any reliable conclusions on potential organ-specific differences in clinical thresholds of epMM loads at this point. In addition, methodological and medical variabilities among studies, such as sample size, endpoint definition, DSA platforms and median fluorescence intensity cutoffs, surveillance/biopsy practices, immunosuppression exposure and adherence, typing resolution and locus inclusion, are further limiting the comparability and interpretation of the existing data.

It has also been shown that immunosuppression exposure, adherence, and surveillance intensity modulate molecular-risk–outcome relationships. Several studies found that subtherapeutic tacrolimus levels in patients with higher HLA class II epMMs were strongly associated with dnDSAs, while recipients with adequate tacrolimus levels rarely developed dnDSAs, even at higher mismatch loads (64–66). Differences in maintenance protocols steroid-sparing vs. conventional) further confound effect estimates across cohorts (64). Assay factors (single-antigen bead positivity thresholds, prozone mitigation) and DSA monitoring schedules vary widely across studies, complicating meta-inference (2, 16).

Transplant urgency could represent another confounder in the interpretation of available evidence on eplet matching in SOT. Lung transplants, like heart and liver, are typically allocated based on urgency, or allocation score, which may come at the cost of worse donor-recipient matching profiles despite a shown reduction in overall waiting-list mortality (67, 68). Study design is also an important source of potential bias. Retrospective cohorts that often rely on imputed high-resolution typing and *post-hoc* epMM calculations are particularly vulnerable to missing data, competing risks, and multiple testing artifacts. Finally, heterogeneity in eplet libraries (and updates) can change the numeric epMM assigned to the same donor–recipient pair, limiting cross-study comparability unless algorithm version and registry snapshot are reported (4). These analytic caveats argue for standardized genotyping, explicit algorithm/version reporting, and similar DSA laboratory thresholds.

Recipient demographics (see Table 1) indicate that most of the work on eplet matching in SOT to-date has been done in males. This aligns with a previous report by Sarah Jackson and colleagues at the National Cancer Institute, that men had received 61.4% of all transplanted organs (69). However, this does not diminish from the fact that a significant portion of transplant recipients are female, and therefore the state of evidence regarding eplet matching in organ transplantation may not be generalizable across gender.

While there is currently no direct evidence comparing epMM counts in SOT across different age groups, reported epMM thresholds were generally lower among pediatric SOT recipients compared to adult recipients. Only two of nine studies reported any epMM thresholds (using HLA Matchmaker) to be >15 among pediatric organ transplant cohorts, while adult-epMM thresholds were highly variable and not-infrequently >20, and in some instances, >30 (10, 70). Notably, however, all pediatric kidney-transplantation articles reporting thresholds defined a cutoff as <15 epMM, and typically <10 epMM. These observations may in part reflect biological differences across age groups, with younger recipients possessing a larger pool of naïve T- and B-cells and heightened thymic activity, which could translate into stronger alloimmune responses even at lower levels of molecular mismatch (71). By contrast, adult recipients are more likely to have experienced prior antigen exposures (e.g., infections, pregnancies, transfusions), shaping memory repertoires and sometimes increasing resilience to new mismatches, overall leading to more variable alloimmune patterns compared with immunologically naïve pediatric recipients (72, 73). Furthermore, the longer expected graft exposure time in pediatric recipients may mean that even modest numbers of epMMs have greater opportunity to manifest as dnDSA and chronic rejection, which also underscores the value of eplet matching for the pediatric transplant population. One included study examining the effect of epMMs in pediatric kidney transplantation found a higher epMM load among different-race donor-recipient pairs as compared to same-race donor-recipient matching, alongside significantly increased risk of rejection predictable by epMM load (33). Of the 98 studies analyzed in this review, this was the only instance of direct, intentional comparison of eplet matching results with graft rejection stratified by race. Considering that the results from Philogene et al. are limited to the pediatric transplant population, future studies should investigate race-specific differences on eplet matching results and their clinical impact across all age groups.

Several studies examined not just the number of epMMs but also their immunogenicity, though definitions vary. Jager et al. identified the ten most immunogenic eplets using a pregnancy model. Bekbolsynov, Kosmoliaptsis, and Lee applied the Cambridge algorithm, while Laux et al. relied on Duquesnoy and Marrari's triplet–serum reactivity correlations. Kleid et al. (17, 74) used ElliPro scores. Findings were mixed: Laux et al. found no association with graft loss, but Kosmoliaptsis, Lee, and Lobashevsky linked immunogenic eplets to dnDSA, and Iwami et al. associated them with AMR in kidney transplantation. Both Kleid studies showed correlations with dnDSA but noted limitations in defining immunogenicity. Importantly, none of the studies compared different immunogenicity definitions, leaving open how methodological choices may influence results.

Of the included five prospective studies four articles examined kidney transplantation, and one was assessing liver transplantation (45, 75–78). Among these studies, four exclusively analyzed adult transplantation cohorts (including three articles on kidney and one on liver transplantation), while one article studied pediatric kidney transplantation. All studies utilized a cohort-based study-sample, four of which received posttransplant triple immunosuppressive therapy; the single study on liver transplantation utilized a single-drug immunosuppressive regimen. All articles examined both Class I and Class II epMMs in association to transplant outcomes. DnDSA development was reported by four studies, TCMR by three, and AMR by two studies. EpMM thresholds significantly associated with all above mentioned adverse transplant outcomes (dnDSA, TCMR, AMR), in addition to graft dysfunction with the exception of Kubal et al., who found that only dnDSA development was significantly related to epMM thresholds in their liver transplant cohort (77). Only two studies had specifically mentioned the use of HLA Matchmaker results to assess donor-recipient compatibility in the organ allocation process (75, 78).

Some articles propose a combined use of the HLAMatchmaker with other HLA epitope mismatch algorithms, most commonly the PIRCHE. HLAMatchmaker and PIRCHE represent complementary immunobiology and different practical footprints. Practically, HLAMatchmaker is widely available at no cost to users (proprietary, free license) and PIRCHE is offered freely for academic use, in addition commercial use with a one-month free trial period after user registration. Recently these practical distinctions have been cataloged alongside related tools: HLA-EMMA (amino-acid mismatch counts emphasizing surface accessibility, free for non-commercial use) and SNOW/Snowflake (deep-learning structural models that weight solvent accessibility and local protrusion, commercial). Together they are a spectrum from empirically curated B-cell epitope surrogates (HLAMatchmaker/EMMA) to physics or computationally informed surface models (SNOW) and T-cell peptide–presentation models (PIRCHE). Recent work indicates that different major versions of PIRCHE produce broadly comparable scores, supporting analytic stability, and allocation simulations in Eurotransplant suggest that incorporating T-cell epitope matching could improve outcomes at the system level (79, 80). Combining B-cell (eplet) and T-cell (PIRCHE) metrics may improve risk stratification; recent reports show independent and additive associations of verified eplet counts, PIRCHE-II, and structural/surface–accessibility indices (SNOW) with dnDSA (7). Alternative structural and surface-accessibility metrics are also being explored to further elucidate risk estimation (7, 81, 82).

What all aformentioned HLA matching algorithms have in common is that they require high-resolution NGS to ensure accurate results. In our review, only ∼16% of studies used NGS; the remainder relied on intermediate or low-resolution methods. Using >61,000 NGS typings, Lhotte et al. showed that inferring high-resolution from intermediate-resolution inputs introduces non-trivial second-field errors, and that these errors translate into false-positive/false-negative eplet assignments. They also note that some tools, like HLA-EMMA, do not impute intermediate-resolution inputs at all, highlighting practical limitations when laboratories type with SSO/SSP and then seek molecular-matching outputs downstream. In a separate NGS-first/SSO-confirmatory cohort, the same group benchmarked imputation directly from intermediate codes and documented accuracy penalties relative to NGS truth sets. Collectively, these data support prioritizing NGS when molecular matching will inform allocation or immunosuppression decisions, while treating SSO/SSP/SSOP/OP-based imputation as a stopgap with explicit uncertainty (83).

Reproducibility relies on curation of “antibody-verified” eplets and on avoiding double counting of overlapping motifs. Systematic appraisal of the Epitope Registry shows that levels of evidence for “verification” vary, and overlapping eplets can inflate counts, affecting thresholds and comparability (4). These concerns are echoed in recent editorial guidance cautioning that overlapping eplets may overestimate mismatch load and urging resolution before threshold-based allocation is contemplated (84).

While higher epMM loads have been associated with increased risk of adverse transplant outcomes at a population level, their predictive value for individual recipients remains limited to date. This is mostly due to the above-mentioned methodical inconsistencies in sequencing and matching processes as well as variabilities in study designs and data collection, but also due to interindividual variability in immune responsiveness, amongst others (88). Current evidence suggests that eplet matching functions primarily as a complementary risk stratification tool rather than a deterministic predictor with potentially strong negative predictive value—patients with very low mismatch counts rarely develop alloimmune injury—yet modest positive predictive value, as many recipients with higher epMM counts do not experience alloimmune consequences (85). This underscores that epMMs reflect immunologic potential rather than inevitable outcome, and that individual results are shaped by additional factors such as immunosuppression exposure, HLA expression, and immune reactivity. The incorporation of standardized high-resolution typing and balanced outcome reporting in future studies will be crucial to improve the accuracy of reported positive predictive epMM thresholds and, in turn, enhance the development of epMM-based personalized risk prediction models. Equally important, future efforts should also define and validate negative predictive thresholds, which at present may offer a more robust and clinically practical approach for integrating eplet matching into donor–recipient compatibility risk stratification.

The question, whether epMM effects are primarily quantitative, reflecting the cumulative impact of multiple small disparities, or qualitative, driven by a few highly immunogenic eplets, remains uncertain. While “antibody-verified” eplets indicate antigenicity, not all elicit clinically significant immune responses, suggesting that many mismatches may be immunologically silent. Higher total epMM loads may therefore increase the likelihood of including one or more truly immunogenic epitopes rather than acting through a purely additive mechanism. The considerable variability in reported thresholds across and within HLA loci likely reflects differences in eplet structure, expression, and accessibility (91). At this stage, the value of this review lies in synthesizing existing data to clarify such patterns, highlight inconsistencies, and identify critical gaps needed to advance toward clinically meaningful epMM-based risk prediction models.

At the bedside, molecular matching has three potential uses: improved donor selection, individualized, risk-stratified immunosuppression tailoring, and patient counseling about risk trade-offs. Observational and interventional work supports using molecular metrics to guide intensity of maintenance therapy, though prospective confirmatory trials remain limited (40, 43, 47). In donor selection, several programs have piloted eplet-aware allocation: living donor networks and at least one pediatric program have incorporated epMM into matching logic, demonstrating feasibility (86).

Allocation policy must balance precision against access. Eurotransplant simulations suggest T-cell epitope matching is implementable without undermining waiting list stability, but there is caution that overlapping eplets, uncertain thresholds, and locus prioritization could inadvertently lengthen waiting times for candidates with rare phenotypes (80, 84). U.S.-based simulation using high-resolution data showed that global 0-DR/DQ epMM targets can reduce disparities relative to 0-ABDR antigen matching, while locus-specific targets may shift disparity patterns (0-DQ favored some groups, 0-DR did not), underscoring the need for prospective equity assessment in any policy (86). In clinical conversations, molecular scores can be used to articulate recipient-specific risk given a particular offer, enabling informed acceptance or deferral by the patient. This could be particularly valuable for non-urgent transplantation, such as kidney transplantation, HSCT, or vascularized composite allotransplantation, while molecular matching appears also highly relevant for the pediatric transplant population. However, the incremental benefit must be weighed against longer waiting and ischemic time risks, especially in organs with narrow ischemia tolerances.

Altogether, the strongest current clinical signal remains at class II: higher DR/DQ epMM associates with dnDSA, AMR, transplant glomerulopathy, and long-term dysfunction across kidney and emerging heart/lung transplant cohorts, while allele-level matching alone shows mixed utility for acute rejection (8, 38). Thoughtful integration of epitope-level metrics appears to be the pragmatic path forward (16, 87).

Three priorities would accelerate clinical translation. First, standardization: routine allele-level genotyping at allocation (including rapid long-read platforms), transparent algorithm/versioning, and harmonized definitions of “antibody-verified” and “immunogenic” eplets (4, 86).

Second, prospective evidence: adequately powered trials and registries across organs that randomize or protocolize decisions informed by molecular scores (immunosuppression titration, donor selection) and report equity outcomes by ancestry. Early randomized and prospective efforts (tacrolimus minimization guided by alloimmunity/mismatch) are instructive but not yet definitive, and multicenter implementation studies are needed (86).

Third, analytics: embrace hybrid predictors that couple B-cell and T-cell pathways with structural features (surface accessibility, physiochemical distances) and validate across diverse ancestries with fixed laboratory thresholds and prespecified endpoints (92). Recent studies demonstrate that multi-predictor models (verified eplets + PIRCHE-II + structural indices) improve dnDSA prediction, a template for precision-medicine deployment (7). Policy development should proceed with simulation and equity impact assessment in lockstep, as recommended by recent allocation analyses and editorials (84, 86). Finally, data reporting would benefit from explicit declaration of typing resolution, software and registry versions, surveillance protocols, and equity analyses, facilitating synthesis across rapidly evolving versions of HLAMatchmaker, PIRCHE, and related tools.

## References

[1] Zachary AA Leffell MS. HLA mismatching strategies for solid organ transplantation—a balancing act. Front Immunol. (2016) 7:575. 10.3389/fimmu.2016.00575 28003816 PMC5141243

[2] Lim WH Wong G Heidt S Claas FHJ. Novel aspects of epitope matching and practical application in kidney transplantation. Kidney Int. (2018) 93(2):314–24. 10.1016/j.kint.2017.08.008 29061333

[3] Wiebe C Nickerson PW. More precise donor-recipient matching: the role of eplet matching. Curr Opin Nephrol Hypertens. (2020) 29(6):630–5. 10.1097/MNH.0000000000000649 32889983

[4] Bezstarosti S Bakker KH Kramer CSM de Fijter JW Reinders MEJ Mulder A A comprehensive evaluation of the antibody-verified Status of eplets listed in the HLA epitope registry. Front Immunol. (2022) 12:800946. 10.3389/fimmu.2021.800946 35154076 PMC8831796

[5] Geneugelijk K Spierings E. PIRCHE-II: an algorithm to predict indirectly recognizable HLA epitopes in solid organ transplantation. Immunogenetics. (2020) 72(1–2):119–29. 10.1007/s00251-019-01140-x 31741009 PMC6971131

[6] Hamada S Dumortier J Thévenin C Pageaux GP Faure S Guillaud O Predictive value of HLAMatchmaker and PIRCHE-II scores for *de novo* donor-specific antibody formation after adult and pediatric liver transplantation. Transpl Immunol. (2020) 61:101306. 10.1016/j.trim.2020.101306 32422222

[7] Chou-Wu E Niemann M Youngs D Gimferrer I. *De novo* donor-specific anti-HLA antibody risk stratification in kidney transplantation using a combination of B cell and T cell molecular mismatch assessment. Front Immunol. (2025) 16:1508796. 10.3389/fimmu.2025.1508796 40070832 PMC11893832

[8] Sapir-Pichhadze R Zhang X Ferradji A Madbouly A Tinckam KJ Gebel HM Epitopes as characterized by antibody-verified eplet mismatches determine risk of kidney transplant loss. Kidney Int. (2020) 97(4):778–85. 10.1016/j.kint.2019.10.028 32059998

[9] Sapir-Pichhadze R Tinckam K Quach K Logan AG Laupacis A John R HLA-DR and -DQ eplet mismatches and transplant glomerulopathy: a nested case–control study. Am J Transplant. (2015) 15(1):137–48. 10.1111/ajt.12968 25521856

[10] Albers EL Friedland-Little JM Hong BJ Kemna MS Warner P Law YM. Human leukocyte antigen eplet mismatching is associated with increased risk of graft loss and rejection after pediatric heart transplant. Pediatr Transplant. (2022) 26(1):e14126. 10.1111/petr.14126 34476876

[11] Sullivan PM Warner P Kemna MS Albers EL Law SP Weiss NS HLA Molecular epitope mismatching and long-term graft loss in pediatric heart transplant recipients. J Heart Lung Transplant. (2015) 34(7):950–7. 10.1016/j.healun.2014.12.017 25727771

[12] Hirama T Akiba M Watanabe T Watanabe Y Oishi H Okada Y. A single-center analysis of how HLA mismatch and donor-specific antibodies affect short-term outcome after lung transplantation: a pilot study before a country-wide histocompatibility study in Japan. Transplant Proc. (2024) 56(2):363–8. 10.1016/j.transproceed.2023.12.011 38320866

[13] Defilippis EM Lacelle C Garg S Farr M. Harnessing precision medicine: HLA or eplet matching in heart transplantation. J Card Fail. (2024) 30(2):373–5. 10.1016/j.cardfail.2023.09.010 37890653

[14] Cardoso B Wang J Kiernan J Dipchand AI. Eplet matching in pediatric heart transplantation: the SickKids experience. J Heart Lung Transplant. (2022) 41(10):1470–7. 10.1016/j.healun.2022.06.023 35933296

[15] Gately R Taverniti A Watson N Teixeira-Pinto A Ooi E Lalji R Comparison between antigen and allelic HLA mismatches, and the risk of acute rejection in kidney transplant recipients. HLA. (2025) 105(4):e70163. 10.1111/tan.70163 40193197 PMC11975158

[16] Tambur AR Das R. Can we use eplets (or molecular) mismatch load analysis to improve organ allocation? The hope and the hype. Transplantation. (2023) 107(3):605–15. 10.1097/TP.0000000000004307 36163639 PMC9944744

[17] Kleid L Walter J Vorstandlechner M Schneider CP Michel S Kneidinger N Predictive value of molecular matching tools for the development of donor specific HLA-antibodies in patients undergoing lung transplantation. HLA. (2023) 102(3):331–42. 10.1111/tan.15068 37068792

[18] Hiho SJ Walton DC Paraskeva MA Levvey BJ Diviney MB Snell GI Determining clinical thresholds for donor HLA eplet compatibility to predict best outcomes following lung transplantation. Transplant Direct. (2022) 8(10):e1364. 10.1097/TXD.0000000000001364 36204183 PMC9529050

[19] Zhang X Kransdorf E Levine R Patel JK Kobashigawa JA. HLA-DQ mismatches stimulate *de novo* donor specific antibodies in heart transplant recipients. Hum Immunol. (2020) 81(7):330–6. 10.1016/j.humimm.2020.04.003 32307126

[20] Delion A Girerd S Duarte K Girerd N Schikowski J Kessler M Which is the best predictor of *de novo* donor-specific antibodies in a cohort of non-sensitized first kidney transplantation: antigenic, allelic, epitope, or physiochemical HLA mismatches? Clin Transplant. (2019) 33(4):e13508. 10.1111/ctr.13508 30821002

[21] Lobashevsky A Goggins W Rosner K Taber T. Immunogenicity of class I HLA but not preformed low MFI donor specific antibodies correlates with outcomes after first renal transplantation. Transpl Immunol. (2017) 43–44:42–8. 10.1016/j.trim.2017.06.001 28629951

[22] Rachisan AL Dubois V Ranchin B Sellier-Leclerc AL Bertholet Thomas A Cochat P Eplet incompatibility in pediatric renal transplantation. Pediatr Transplant. (2020) 24(6):e13721. 10.1111/petr.13721 32388894

[23] Ashimine S Sakamoto S Tomosugi T Spierings E Niemann M Shizuku M Which is more important for predicting *de novo* DSA production in donor-sensitized kidney transplant recipients, B-cell epitope or T-cell epitope analysis? Hum Immunol. (2024) 85(6):111155. 10.1016/j.humimm.2024.111155 39536340

[24] Snanoudj R Kamar N Cassuto E Caillard S Metzger M Merville P Epitope load identifies kidney transplant recipients at risk of allosensitization following minimization of immunosuppression. Kidney Int. (2019) 95(6):1471–85. 10.1016/j.kint.2018.12.029 30955869

[25] Lachmann N Niemann M Reinke P Budde K Schmidt D Halleck F Donor-recipient matching based on predicted indirectly recognizable HLA epitopes independently predicts the incidence of *de novo* donor-specific HLA antibodies following renal transplantation. Am J Transplant. (2017) 17(12):3076–86. 10.1111/ajt.14393 28613392

[26] Wen J Basu A Bentall A Henderson N Dukek B Gandhi M Is the level of HLA eplet mismatch a risk factor for graft loss among kidney transplant recipients who have already formed *de novo* donor specific antibody? Hum Immunol. (2021) 82(4):240–6. 10.1016/j.humimm.2021.02.004 33618904

[27] Do Nguyen HT Wong G Chapman JR McDonald SP Coates PT Watson N The association between broad antigen HLA mismatches, eplet HLA mismatches and acute rejection after kidney transplantation. Transplant Direct. (2016) 2(12):e120. 10.1097/TXD.0000000000000632 27990485 PMC5142364

[28] Laux G Mytilineos J Opelz G. Critical evaluation of the amino acid triplet-epitope matching concept in cadaver kidney transplantation. Transplantation. (2004) 77(6):902–7. 10.1097/01.TP.0000114595.59168.3B 15077035

[29] Walton DC Hiho SJ Cantwell LS Diviney MB Wright ST Snell GI HLA matching at the eplet level protects against chronic lung allograft dysfunction. Am J Transplant. (2016) 16(9):2695–703. 10.1111/ajt.13798 27002311

[30] Yanyiam P Kantachuvesiri S Thammanichanond D. Impact of HLA eplet mismatch on *de novo* donor specific antibody formation after kidney transplantation. Transplant Proc. (2024) 56(3):515–20. 10.1016/j.transproceed.2024.01.030 38368130

[31] Alves TA Nascimento E de Castro LB Fabreti-Oliveira RA. Impact of HLA eplet mismatch load in immunological outcomes after living donor kidney transplantation. Transpl Immunol. (2023) 80:101908. 10.1016/j.trim.2023.101908 37536379

[32] Tafulo S Malheiro J Santos S Dias L Almeida M Martins S Degree of HLA class II eplet mismatch load improves prediction of antibody-mediated rejection in living donor kidney transplantation. Hum Immunol. (2019) 80(12):966–75. 10.1016/j.humimm.2019.09.010 31604581

[33] Philogene MC Amin A Zhou S Charnaya O Vega R Desai N Eplet mismatch analysis and allograft outcome across racially diverse groups in a pediatric transplant cohort: a single-center analysis. Pediatr Nephrol. (2020) 35(1):83–94. 10.1007/s00467-019-04344-1 31599339 PMC6901410

[34] Silva E Alba A Castro A Carrascal M Buckel E Aguilo J Evaluation of HLA matchmaker compatibility as predictor of graft survival and presence of anti-HLA antibodies. Transplant Proc. (2010) 42(1):266–9. 10.1016/j.transproceed.2009.12.047 20172326

[35] Sakamoto S Iwasaki K Tomosugi T Niemann M Spierings E Miwa Y Analysis of T and B cell epitopes to predict the risk of *de novo* donor-specific antibody (DSA) production after kidney transplantation: a two-center retrospective cohort study. Front Immunol. (2020) 11:2000. 10.3389/fimmu.2020.02000 32973806 PMC7481442

[36] Lee H Min JW Kang H Lee H Eum SH Park Y Combined analysis of HLA class II eplet mismatch and tacrolimus levels for the prediction of *de novo* donor specific antibody development in kidney transplant recipients. Int J Mol Sci. (2022) 23(13):4–10. 10.3390/ijms23137357 PMC9267119 35806362

[37] Kishikawa H Kinoshita T Hashimoto M Fukae S Taniguchi A Yamanaka K Class II HLA eplet mismatch is a risk factor for *de novo* donor-specific antibody development and antibody-mediated rejection in kidney transplantation recipients. Transplant Proc. (2018) 50(8):2388–91. 10.1016/j.transproceed.2018.02.183 30316363

[38] Wiebe C Pochinco D Blydt-Hansen TD Ho J Birk PE Karpinski M Class II HLA epitope matching—a strategy to minimize *de novo* donor-specific antibody development and improve outcomes. Am J Transplant. (2013) 13(12):3114–22. 10.1111/ajt.12478 24164958

[39] Wiebe C Nevins TE Robiner WN Thomas W Matas AJ Nickerson PW. The synergistic effect of class II HLA epitope-mismatch and nonadherence on acute rejection and graft survival. Am J Transplant. (2015) 15(8):2197–202. 10.1111/ajt.13341 26095765

[40] Tran J Alrajhi I Chang D Sherwood KR Keown P Gill J Clinical relevance of HLA-DQ eplet mismatch and maintenance immunosuppression with risk of allosensitization after kidney transplant failure. Front Genet. (2024) 15:1383220. 10.3389/fgene.2024.1383220 38638120 PMC11024336

[41] Daniëls L Naesens M Bosmans JL Abramowicz D Nagler E Van Laecke S The clinical significance of epitope mismatch load in kidney transplantation: a multicentre study. Transpl Immunol. (2018) 50:55–9. 10.1016/j.trim.2018.06.006 29908316

[42] Maguire C Crivello P Fleischhauer K Isaacson D Casillas A Kramer CSM Qualitative, rather than quantitative, differences between HLA-DQ alleles affect HLA-DQ immunogenicity in organ transplantation. HLA. (2024) 103(4):e15455. 10.1111/tan.15455 38575370 PMC11003724

[43] Bezstarosti S Meziyerh S Reinders MEJ Voogt-Bakker K Groeneweg KE Roelen DL HLA-DQ eplet mismatch load may identify kidney transplant patients eligible for tacrolimus withdrawal without donor-specific antibody formation after mesenchymal stromal cell therapy. HLA. (2023) 102(1):3–12. 10.1111/tan.15008 36841928

[44] San Segundo D Guiral-Foz SA Benito-Hernandez A Fernandez AR Arnau A Valero R Urinary CXCL10 specifically relates to HLA-DQ eplet mismatch load in kidney transplant recipients. Transpl Immunol. (2022) 70:101494. 10.1016/j.trim.2021.101494 34774739

[45] Bestard O Meneghini M Crespo E Bemelman F Koch M Volk HD Preformed T cell alloimmunity and HLA eplet mismatch to guide immunosuppression minimization with tacrolimus monotherapy in kidney transplantation: results of the CELLIMIN trial. Am J Transplant. (2021) 21(8):2833–45. 10.1111/ajt.16563 33725408

[46] Jager C Niemann M Honger G Wehmeier C Hopfer H Menter T Combined molecular mismatch approaches to predict immunological events within the first year after renal transplantation. HLA. (2024) 104(5):e15748. 10.1111/tan.15748 39501813 PMC11586251

[47] Johnson AC Zhang J Karadkhele G Gragert L Hertzberg V Larsen CP. Belatacept with time-limited tacrolimus coimmunosuppression modifies the 3-year risk of eplet mismatch in kidney transplantation. Am J Transplant. (2024) 24(2):260–70. 10.1016/j.ajt.2023.09.011 37778459 PMC10842047

[48] Wong ETY Pochinco D Vathsala A Koh WK Lim A Sran HK HLA-DR/DQ eplet mismatch predicts *de novo* donor-specific antibody development in multi-ethnic southeast Asian kidney transplant recipients on different immunosuppression regimens. Front Genet. (2024) 15:1447141. 10.3389/fgene.2024.1447141 39262421 PMC11387181

[49] Wiebe C Kosmoliaptsis V Pochinco D Gibson IW Ho J Birk PE HLA-DR/DQ molecular mismatch: a prognostic biomarker for primary alloimmunity. Am J Transplant. (2019) 19(6):1708–19. 10.1111/ajt.15177 30414349 PMC6563434

[50] Rampersad C Wiebe C Balshaw R Bullard J Gibson IW Trachtenberg A Association of BKV viremia and nephropathy with adverse alloimmune outcomes in kidney transplant recipients. Clin Transplant. (2024) 38(5):e15329. 10.1111/ctr.15329 38722085

[51] Smith J Dharnidharka V. Progress in pediatric kidney transplantation. Open Urol Nephrol J. (2014) 7:115–22. 10.2174/1874303X014070100115 PMC4366268 25821528

[52] Poggio ED Augustine JJ Arrigain S Brennan DC Schold JD. Long-term kidney transplant graft survival-making progress when most needed. Am J Transplant. (2021) 21(8):2824–32. 10.1111/ajt.16463 33346917

[53] Murdoch B Wagner D Sandal S Sherwood K. The rule of rescue in the era of precision medicine, HLA eplet matching, and organ allocation. Can J Bioeth. (2023) 6(2):36–42. 10.7202/1101126ar

[54] Tiercy JM. How to select the best available related or unrelated donor of hematopoietic stem cells? Haematologica. (2016) 101(6):680–7. 10.3324/haematol.2015.141119 27252513 PMC5013969

[55] De Santis D Truong L Martinez P D'Orsogna L. Rapid high-resolution HLA genotyping by MinION Oxford nanopore sequencing for deceased donor organ allocation. HLA. (2020) 96(2):141–62. 10.1111/tan.13901 32274854

[56] Devriese M Da Silva S Le Mene M Rouquie J Allain V Kolesar L Two-field resolution on-call HLA typing for deceased donors using nanopore sequencing. HLA. (2024) 103(3):e15441. 10.1111/tan.15441 38507216

[57] Wang J Wang P Wang S Tan J. Donor-specific HLA antibodies in solid organ transplantation: clinical relevance and debates. Explor Res Hypothesis Med. (2019) 4(4):76–86. 10.14218/ERHM.2019.00012

[58] Meneghini M Tambur AR. HLA-DQ antibodies in alloimmunity, what makes them different? Curr Opin Organ Transplant. (2023) 28(5):333–9. 10.1097/MOT.0000000000001079 37219535 PMC10487393

[59] Hirahara K Kokubo K Aoki A Kiuchi M Nakayama T. The role of CD4+ resident memory T cells in local immunity in the mucosal tissue—protection versus pathology. Front Immunol. (2021) 12:616309. 10.3389/fimmu.2021.616309 33968018 PMC8097179

[60] Nguyen QP Deng TZ Witherden DA Goldrath AW. Origins of CD4(+) circulating and tissue-resident memory T-cells. Immunology. (2019) 157(1):3–12. 10.1111/imm.13059 30897205 PMC6459775

[61] Phillips BL Callaghan C. The immunology of organ transplantation. Surgery (Oxford). (2017) 35(7):333–40. 10.1016/j.mpsur.2017.04.004

[62] Madariaga ML Kreisel D Madsen JC. Organ-specific differences in achieving tolerance. Curr Opin Organ Transplant. (2015) 20(4):392–9. 10.1097/MOT.0000000000000206 26147678 PMC4523268

[63] Jiang X Nicolls MR. Working toward immune tolerance in lung transplantation. J Clin Invest. (2014) 124(3):967–70. 10.1172/JCI74701 24569371 PMC3938269

[64] Niemann M Matern BM. Molecular matching tools for allocation and immunosuppression optimization. Ready for primetime? Curr Opin Organ Transplant. (2025) 30(1):30–6. 10.1097/MOT.0000000000001185 39711242 PMC11676598

[65] Wiebe C Rush DN Nevins TE Birk PE Blydt-Hansen T Gibson IW Class II eplet mismatch modulates tacrolimus trough levels required to prevent donor-specific antibody development. J Am Soc Nephrol. (2017) 28(11):3353–62. 10.1681/ASN.2017030287 28729289 PMC5661295

[66] Davis S Wiebe C Campbell K Anobile C Aubrey M Stites E Adequate tacrolimus exposure modulates the impact of HLA class II molecular mismatch: a validation study in an American cohort. Am J Transplant. (2021) 21(1):322–8. 10.1111/ajt.16290 32888256 PMC7821185

[67] Egan TM Edwards LB. Effect of the lung allocation score on lung transplantation in the United States. J Heart Lung Transplant. (2016) 35(4):433–9. 10.1016/j.healun.2016.01.010 26922274

[68] Valapour M Lehr CJ Wey A Skeans MA Miller J Lease ED. Expected effect of the lung composite allocation score system on US lung transplantation. Am J Transplant. (2022) 22(12):2971–80. 10.1111/ajt.17160 35870119

[69] Jackson SS Pfeiffer RM Hsieh MC Li J Madeleine MM Pawlish KS Sex differences in cancer incidence among solid organ transplant recipients. J Natl Cancer Inst. (2024) 116(3):401–7. 10.1093/jnci/djad224 37944040 PMC10919340

[70] Shin S Lee M Dente E Yazigi N Khan Khalid M Kaufman SS Mismatch epitope load predicts *de novo*-DSA-free survival in pediatric liver transplantation. Pediatr Transplant. (2022) 26(4):e14251. 10.1111/petr.14251 35279919

[71] Baghai Arassi M Feißt M Krupka K Awan A Benetti E Düzova A Age-related differences in rejection rates, infections, and tacrolimus exposure in pediatric kidney transplant recipients in the CERTAIN registry. Kidney Int Rep. (2024) 9(11):3265–77. 10.1016/j.ekir.2024.08.025 39534206 PMC11551099

[72] Porrett PM. Biologic mechanisms and clinical consequences of pregnancy alloimmunization. Am J Transplant. (2018) 18(5):1059–67. 10.1111/ajt.14673 29369525

[73] Willicombe M Roberts DJ. Transfusion-induced HLA sensitization in wait-list patients and kidney transplant recipients. Kidney Int. (2024) 106(5):795–805. 10.1016/j.kint.2024.07.030 39181398

[74] Kleid L Walter J Moehnle P Wichmann C Kovács J Humpe A High-risk HLA-DQ mismatches are associated with adverse outcomes after lung transplantation. Transpl Int. (2024) 37:13010. 10.3389/ti.2024.13010 39381015 PMC11460317

[75] Kausman JY Walker AM Cantwell LS Quinlan C Sypek MP Ierino FL. Application of an epitope-based allocation system in pediatric kidney transplantation. Pediatr Transplant. (2016) 20(7):931–8. 10.1111/petr.12815 27662811

[76] Diebold M Vietzen H Schatzl M Mayer KA Haindl S Heinzel A Functional natural killer-cell genetics and microvascular inflammation after kidney transplantation: an observational cohort study. Transplantation. (2025) 109(5):860–70. 10.1097/TP.0000000000005228 39402708 PMC12011434

[77] Kubal CA Mangus R Ekser B Mihaylov P Ceballos B Higgins N Class II human leukocyte antigen epitope mismatch predicts *de novo* donor-specific antibody formation after liver transplantation. Liver Transpl. (2018) 24(8):1101–8. 10.1002/lt.25286 30142248

[78] González-López E Ocejo-Vinyals JG Renuncio-García M Roa-Bautista A San Segundo Arribas D Escagedo C Donor-derived cell-free DNA at 1 month after kidney transplantation relates to HLA class II eplet mismatch load. Biomedicines. (2023) 11(10):2741. 10.3390/biomedicines11102741 37893114 PMC10604614

[79] Matern BM Niemann M. PIRCHE Application major versions 3 and 4 lead to equivalent T cell epitope mismatch scores in solid organ and stem cell transplantation modules. Hum Immunol. (2024) 85(3):110789. 10.1016/j.humimm.2024.110789 38521663

[80] Niemann M Lachmann N Geneugelijk K Spierings E. Computational eurotransplant kidney allocation simulations demonstrate the feasibility and benefit of T-cell epitope matching. PLoS Comput Biol. (2021) 17(7):e1009248. 10.1371/journal.pcbi.1009248 34314431 PMC8345832

[81] Kim JJ Fichtner A Copley HC Gragert L Süsal C Dello Strologo L Molecular HLA mismatching for prediction of primary humoral alloimmunity and graft function deterioration in paediatric kidney transplantation. Front Immunol. (2023) 14:1092335. 10.3389/fimmu.2023.1092335 37033962 PMC10080391

[82] de Marco R Requião-Moura LR Raimundo TRF Mourão TB Rampim GF Medina-Pestana JO HLA-DPB1 molecular mismatches are risk factors for acute rejection and low 5-year graft function in first kidney transplants. HLA. (2023) 101(3):228–38. 10.1111/tan.14911 36461794

[83] Lhotte R Letort V Usureau C Jorge-Cordeiro D Siemowski J Gabet L Improving HLA typing imputation accuracy and eplet identification with local next-generation sequencing training data. HLA. (2024) 103(1):e15222. 10.1111/tan.15222 38589051

[84] Karahan GE Haasnoot GW Heidt S. Equitable allocation through human leukocyte antigen eplet matching: a promising strategy with several challenges. Am J Transplant. (2025) 25(6):1149–51. 10.1016/j.ajt.2025.01.028 39855343

[85] Senev A Coemans M Lerut E Van Sandt V Kerkhofs J Daniëls L Eplet mismatch load and *de novo* occurrence of donor-specific anti-HLA antibodies, rejection, and graft failure after kidney transplantation: an observational cohort study. J Am Soc Nephrol. (2020) 31(9):2193–204. 10.1681/ASN.2020010019 32764139 PMC7461684

[86] Mankowski MA Gragert L Keating B Lonze BE Segev DL Montgomery R Balancing equity and human leukocyte antigen matching in deceased-donor kidney allocation with eplet mismatch. Am J Transplant. (2025) 25(6):1226–34. 10.1016/j.ajt.2024.11.030 39631566

[87] Duquesnoy RJ. Are we ready for epitope-based HLA matching in clinical organ transplantation? Transplantation. (2017) 101(8):1755–65. 10.1097/TP.0000000000001667 28207632

[88] Böhringer D Daub F Schwartzkopff J Maier P Birnbaum F Sundmacher R Operational post-keratopasty graft tolerance due to differential HLAMatchmaker matching. Mol Vis. (2010) 16:2362–7. 21152438 PMC2994735

[89] Daniëls L Claas FHJ Kramer CSM Senev A Vanden Driessche M Emonds M-P The role of HLA-DP mismatches and donor specific HLA-DP antibodies in kidney transplantation: a case series. Transpl Immunol. (2021) 65:101287. 10.1016/j.trim.2020.101287 32194154

[90] Duquesnoy RJ Marrari M. Detection of antibodies against HLA-C epitopes in patients with rejected kidney transplants. Transpl Immunol. (2011) 24(3):164–71. 10.1016/j.trim.2010.12.003 21185937

[91] Mohammadhassanzadeh H Oualkacha K Zhang W Klement W Bourdiec A Lamsatfi J On path to informing hierarchy of eplet mismatches as determinants of kidney transplant loss. Kidney Int Rep. (2021) 6(6):1567–79. 10.1016/j.ekir.2021.03.877 34169197 PMC8207474

[92] Chaigne B Geneugelijk K Bédat B Ahmed MA Hönger G De Seigneux S Immunogenicity of anti-HLA antibodies in pancreas and islet transplantation. Cell Transplant. (2016) 25(11):2041–50. 10.3727/096368916X691673 27196533

