# Supplementary material for: The role of eplet matching in solid organ transplantation
Source: Front Transplant. 2025 Dec 8;4:1710058. doi: 10.3389/frtra.2025.1710058 (PMC12719434; doi:10.3389/frtra.2025.1710058)
Supplement: Supplementary file 1 [file Table1.docx]

| Study | Transplanted Organ/Tissue | Epitope Matching Method | *n* (recipients) | Recipient Age Group | HLA Loci Assessed | Loci Associated with Outcomes | Mismatch Thresholds | HLA Matching for Threshold | Predictive outcome of threshold |
| --- | --- | --- | --- | --- | --- | --- | --- | --- | --- |
| Albers et al., 2021 | Heart | HLA Matchmaker | 1,887 | Pediatric | A, B, DR | A, B, DR | Class I epMM (Low risk < 10, Medium risk 10-20, High risk >20), Class II (Low risk <10, Medium risk 10-20, High risk >20), Alleles Class II (0-1, 2, 3, 4), Alleles Class II (0, 1, 2) | HLA Matchmaker | dnDSA, Rejection |
| Aldea et al., 2023 | Kidney | HLA Matchmaker, PIRCHE | 55 | Pediatric | A, B, DR | - | Low risk: <4 or High risk: ≥ 4 | HLA Matchmaker, PIRCHE | - |
| Alves et al., 2023 | Kidney | HLA Matchmaker | 159 | Adult | A, B, DRB1 | DR | A/B Low risk: 0-16, A/B Medium risk: 17-29, A/B High risk: >29; DR Low risk: 0-10, DR Medium risk: 11-20, DR High risk: >20 | HLA Matchmaker | AMBR, TCMR, Generalized rejection |
| Arches et al., 2024 | Kidney | HLA Matchmaker | 45 | Adult | A, B, Cw, DRB1, DRB3/4/5, DQB1/A1 | Cw | Low risk: 0 EpMM, Medium-low risk: 1-3 EpMM, Medium-high risk: 4-6 EpMM, High risk: ≥ 7 EpMM | HLA Matchmaker | dnDSA, TCMR |
| Ashimine et al., 2024 | Kidney | HLA Matchmaker, PIRCHE | 112 | Adult | A, B, DRB1, DQA1/B1 | DQA/B, DR | 8 eplet mismatch (exp), 5 eplet mismatch (Ctrl) for dnDSA production; graft survival 0, 1-7, 8-25 mismatches or 0, 1-4, 5-25 | HLA Matchmaker, PIRCHE | dnDSA |
| Bekbolsynov et al., 2020 | Kidney | HLA Matchmaker | 78,864 | Adult | A, B, DRB1, DQB1 | DR, DQ | - | - | Graft loss |
| Bestard et al., 2021 | Kidney | HLA Matchmaker | 167 | Adult | A, B, C, DRB1, DQA1/B1 | DRB1, DQ | DQA1/B1 ≥ 10, DQ Low risk: 0, Medium risk: 1-5, High risk: ≥ 6 | HLA Matchmaker | dnDSA, ABMR, TCMR |
| Bezstarosti et al., 2023 | Kidney | HLA Matchmaker | 70 | Adult | A, B, DR, DQ | Class II, DQ | ≥ 11 (DQ) | HLA Matchmaker | dnDSA |
| Bosch et al., 2012 | Kidney | HLA Matchmaker | 1 | Adult | A, B | 62QE | - | HLA Matchmaker | - |
| Bosch et al., 2014 | Kidney | HLA Matchmaker | 1 | Adult | A, B, C, DRB1, DQB1 | - | - | - | - |
| Cardoso et al., 2022 | Heart | HLA Matchmaker | 77 | Pediatric | A, B, C, DRB1, DRB 3/4/5, DQA1, DQB1, DPA, DPB | DPB | ≥2 epMM (DPB) | HLA Matchmaker | Graft loss |
| Charnaya et al., 2021 | Kidney | HLA Matchmaker | 125 | Pediatric | A, B, C, DRB1, DQB1 | Class I | - | - | - |
| Chou-Wu et al., 2025 | Kidney | HLA Matchmaker, PIRCHE, SNOW | 843 | Mixed | A, B, C, DRB1, DQB1 | Class II > Class I | >8 Class I, >3 Class II | HLA Matchmaker | dnDSA |
| Crespo et al., 2021 | Kidney | HLA Matchmaker | 118 | Adult | A, B, C, DR, DQ | Class II (notably DRB1) | - | - | dnDSA |
| Daniels et al., 2018 | Kidney | HLA Matchmaker, PIRCHE | 36 | Mixed | A, B, DR, DQ | A, B, C, DR, DQ | DQB1 ≥ 6 | HLA Matchmaker | dnDSA |
| Daniels et al., 2020 | Kidney | HLA Matchmaker | 86 | Mixed | DP | - | - | - | dnDSA |
| de Marco et al., 2023 | Kidney | HLA Matchmaker, HLA-EMMA, TCE Algorithm | 130 | Pediatric | DPB1 | DPB1 | - | - | - |
| de Marco et al., 2025 | Kidney | HLA Matchmaker, PIRCHE, HLA-EMMA | 198 | Mixed | A, B, C, DRB1/345, DQA1/B1, DPA1/B1 | HLA-C, HLA-DQA1/B1, HLA-DQB1 | - | HLA Matchmaker, PIRCHE, HLA-EMMA | - |
| Delion et al., 2019 | Kidney | HLA Matchmaker | 150 | Adult | A, B, C, DR, DQ | - | > 22 epMM | HLA Matchmaker | dnDSA |
| Demir et al., 2025 | Kidney | HLA Matchmaker, PIRCHE | 5,159 | Adult | A, B, C, DQB1, DRB1 | Class II | - | - | - |
| Diebold et al., 2025 | Kidney | HLA Matchmaker | 507 | Adult | A, B, C, DR, DQ, DP | A, B, C | - | - | Graft dysfunction |
| Duquesnoy, 2010 | Kidney | HLA Matchmaker | 45 | - | C | - | 11 epMM for >10% MFI, 7 epMM for <10% MFI (MFI = median fluorescence intensity) | HLA Matchmaker | dnDSA |
| Ekong et al., 2019 | Liver | HLA Matchmaker | 42 | Pediatric | A, B, C, DRB1/3/4/5, DQA/B, DPA/B | DQ | ≥ 6 (DQ); unable to define a threshold for DR | HLA Matchmaker | dnDSA, TCMR |
| Forner et al., 2018 | Liver | HLA Matchmaker | 79 | Adult | A, B, C, DRB1, DQA/B |  | - | - | AMBR, TCMR, Recipient survival |
| Gao et al., 2023 | Kidney | HLA Matchmaker | 526 | Adult | A, B, C, DRB1, DQA1/B1 | B | - | - | dnDSA, AMBR, TCMR |
| Guiral et al., 2020 | Liver | HLA Matchmaker | 43 | Adult | A, B, C, DR, DQ | C | VerEpC <2 or >=2 | HLA Matchmaker | TCMR |
| Hamada et al., 2020 | Liver | HLA Matchmaker, PIRCHE | 540 | Mixed | A, B, C, DR, DQ, DP | Adult: all epMM, Pediatric: Class II | - | - | - |
| Hiho et al., 2022 | Lung | HLA Matchmaker | 277 | Adult | A, B, C, DRB1/3/4/5, DQA1/B1, DPA1/B1 | Class II, DRB1/3/4/5, DQA1 | class II ≥ 19, Classes I and II combined ≥29 | HLA Matchmaker | Graft dysfunction |
| Hiho et al., 2024 | Lung | HLA Matchmaker, PIRCHE, HLA-EMMA | 277 | Adult | A, B, C, DRB1/3/4/5, DQA1/B1, DPA1/B1 | DR, DQ | - | HLA Matchmaker, PIRCHE, HLA-EMMA | dnDSA, Graft dysfunction |
| Hirama et al., 2024 | Lung | HLA Matchmaker | 45 | Adult | A, B, C, DRB1, DQB1 | - | ≥ 60 | HLA Matchmaker | TCMR, Graft dysfunction |
| Hung Thanh Do Nguyen et al., 2016 | Kidney | HLA Matchmaker | 3,449 | Adult | A, B, DR | A, B, DR | HLA-ABDR 0-2, 3-10, 11-20, >20 | HLA Matchmaker | TCMR |
| Iwami et al., 2017 | Kidney | HLA Matchmaker | 55 | Mixed | A, B, DRB1, DQB1 | DQB1 | - | - | - |
| Jabbour et al., 2024 | Kidney | HLA Matchmaker, PIRCHE, HLA-EMMA | 117 | Adult | A, B, C, DRB1/3/4/5, DQA1/B1 | DQB1 | - | - | - |
| Jager et al., 2024 | Kidney | HLA Matchmaker, PIRCHE | 439 | Adult | A, B, C, DR, DQ, DP | - | Low risk: total epMM < 73 + "Top 10 immunogenic eplets" mismatch < 4 or PIRCHEII < 93; High risk: total epMM ≥ 73 + "Top 10 immunogenic eplets" mismatch > 4 or PIRCHEII ≥ 93 | HLA Matchmaker, PIRCHE | AMBR, TCMR |
| Johnson et al., 2024 | Kidney | HLA Matchmaker | 594 | Adult | DR, DQ | DR, DQ | Low risk: DR < 7 epMM and DQ < 9 epMM; Intermediate risk: DR ≥ 7 epMM or DQ between 9-14 EpMM; High risk: DQ ≥ 15 epMM | HLA Matchmaker | dnDSA, ABMR |
| Kamoun et al., 2025 | Kidney | HLA Matchmaker, PIRCHE | 112 | Adult | A, B, DRB1, DQA1, DQB1 | DQ | - | - | Graft loss |
| Kausman et al., 2016 | Kidney | HLA Matchmaker | 19 | Pediatric | C, DQA1/B1 | Class II | - | - | dnDSA |
| Kishikawa et al., 2018 | Kidney | HLA Matchmaker | 167 | Adult | A, B, C, DRB1/3/4/5, DQA/B | DRB1/3/4/5, DQA/B | DRB1/3/4/5 eplet MM<15, >=15; DQA/B eplet MM<15, >=15 (ABMR), eplet MM >5, <=5 DSA | HLA Matchmaker | dnDSA, ABMR |
| Kleid et al., 2023 | Lung | HLA Matchmaker, PIRCHE | 183 | Adult | A, B, C, DRB1/3/4/5, DQA1/B1, DPA1/B1 | A, highly immunogenic MM | Class II DSA: 42.5 epMM CO, 30.5 immunogenic epMM CO, 85.50 PIRCHE-II (5), 560.00 PIRCHE-II (11); DQ DSA 17.50 epMM CO, 13.50 immunogenic epMM | HLA Matchmaker, PIRCHE | dnDSA |
| Kleid et al., 2024 | Lung | HLA Matchmaker | 183 | Adult | A, B, C, DRB1/3/4/5, DQA1/B1, DPA1/B1 | DQ | - | - | dnDSA, AMBR |
| Kok et al., 2022 | Liver | HLA Matchmaker, PIRCHE | 736 | Adult | - | - | - | - | - |
| Kosmoliaptsis et al., 2016 | Kidney | HLA Matchmaker | 131 | Adult | A, B, C, DRB1/3/4/5, DQ | - | - | HLA Matchmaker | dnDSA |
| Kubal et al., 2016 | Pancreas | HLA Matchmaker, PIRCHE | 44 | Adult | A, B, DRB1. DQB1 | - | - | - | - |
| Kubal et al., 2018 | Liver | HLA Matchmaker | 80 | Adult | A, B, C, DRB1/3/4/5, DQA/B | Class II, Class I | - | - | dnDSA |
| Lachmann et al., 2017 | Kidney | HLA Matchmaker, PIRCHE | 2,787 | Adult | A, B, C, DR, DQ | DRB, DQB | epMM Low risk: <5, epMM Medium-low risk: ≥5 to <18, epMM Medium-high risk: ≥18 to <36, epMM High risk: ≥ 36; PIRCHE Low risk: <9, PIRCHE Medium-low risk: ≥ 9 to <35, PIRCHE Medium-high risk: ≥ 35 to <90, PIRCHE High risk: ≥ 90 | HLA Matchmaker, PIRCHE | dnDSA, Graft loss |
| Larkins et al., 2022 | Kidney | HLA Matchmaker | 145 | Pediatric | A, B, C, DRB1/3/4/5, DQA1/B1, DPA1/B1 | DRB1, DQA1, DQB1 | - | - | - |
| Laux et al., 2004 | Kidney | HLA Matchmaker | 16,997 | - | A, B, DR | A, B | Low risk: 0-6, Medium-low risk: 7-9, Medium-high risk: 10-12, High risk: ≥13 triplet epMM; Low risk: 0-5, Medium-low risk: 6-8, Medium-high risk: 9-11, High-risk: ≥12 immunogenic triplet MM | HLA Matchmaker | - |
| Lee et al., 2022 | Kidney | HLA Matchmaker | 347 | Adult | DRB1, DQA1/B1 | - | Single mismatch analysis >7 DR, >9 DQ, total mismatch analysis: DR > 13, DQ > 9; antibody verified mismatch DR > 2, DQ > 1; antibody verified single molecular DR > 2, DQ > 1 | HLA Matchmaker | dnDSA, ABMR |
| Li et al., 2025 | Kidney | HLA Matchmaker | 64 | Adult | A, B, C, DRB1, DQA1/B1 | A, B | - | - | - |
| Liu et al., 2022 | Kidney | HLA Matchmaker | 1 | Adult | - | DQB1*3:03 | - | - | - |
| Lobashevsky et al., 2017 | Kidney | HLA Matchmaker | 41 | Adult | A, B, C, DRB, DQ | A | >12 (all) | HLA Matchmaker | Adverse medical/surgical events |
| Lopez et al., 2023(a) | Kidney | HLA Matchmaker | 42 | Adult | "Class I", DRB1, DQA1/B1 | DQB1 | - | - | Donor-derived cell-free DNA |
| Lopez et al., 2023(b) | Lung | HLA Matchmaker | 240 | Adult | A, B, C, DR, DQ | DQA1 | - | - | Graft dysfunction |
| Maguire et al., 2024 | Kidney | HLA Matchmaker | 279 | Adult | DQA1/B1 | DQ | > 8, Class 11 | Youden's Score | dnDSA |
| Mangiola et al., 2022 | Heart | HLA Matchmaker | 274 | Pediatric | "Class I"; DRB1/3/4/5; DQA1/B1 | Class I, DR, DQ | Class I epMM 13(DSA), 9 (ABMR); Class I PIRCHE: 141 (DSA), 157 (AMBR); Class II epMM 9 (DSA), Class II PIRCHE:80 (DSA), 116 (AMBR) | PIRCHE | dnDSA, ABMR |
| Marrari & Duqesnoy, 2009 | Kidney | HLA Matchmaker | 19 | Adult | DRB1/3/4/5 | DRB1 | - | - | - |
| Marrari et al., 2011 | Kidney | HLA Matchmaker | 2 | Adult | A, B, C | - | - | - | - |
| McCaughan et al., 2018 | Heart | HLA Matchmaker | 264 | Adult | A, B, C, DRB1/3/4/5, DQA1/B1 | DQ | - | - | - |
| Meneghini et al., 2018 | Kidney | HLA Matchmaker | 330 | Adult | A, B, DR, DQ | DR, DQ | - | - | - |
| Meneghini et al., 2021 | Kidney | HLA Matchmaker, PIRCHE | 169 | Adult | A, B, C, DRB1, DQB1, DPB1 | DQB1, DRB1 | 50 | PIRCHE | TCMR |
| Nascimento et al., 2016 | Kidney | HLA Matchmaker | 1 | Adult | A, B, C, DQA1, DPA1/B1, MIC-A | DPB1 | - | - | - |
| Nilsson et al., 2019 | Heart | HLA Matchmaker | 34,681 | Adult | A, B, C, DRB1/3/4/5, DQA1/B1, DPB1 | DR/DQ | - | - | - |
| Osorio-Jaramillo et al., 2020 | Heart | HLA Matchmaker, HLA-EMMA | 1,167 | Adult | A, B, DR | DR | - | - | - |
| Otten et al., 2013 | Kidney | HLA Matchmaker, PIRCHE | 21 | - | A, B, DR | A, B, DR | - | - | - |
| Philogene et al., 2020 | Kidney | HLA Matchmaker | 110 | Pediatric | A, B, C, DR, DQ, DP | DR/DQ | >70 | HLA Matchmaker | ABMR, TCMR |
| Pouliquen et al., 2017 | Pancreas | HLA Matchmaker | 42 | Adult | - | - | - | - | - |
| Rãchişan et al., 2020 | Kidney | HLA Matchmaker | 70 | Pediatric | A, B, C, DR, DQ | DR/DQ | ≥ 16 | HLA Matchmaker | dnDSA |
| Rampersad et al., 2024 | Kidney | HLA Matchmaker | 460 | Mixed | DRB1/3/4/5, DQA1/B1 | - | Low RAMM (age younger than 35 and low epMM), intermediate RAMM (35 years or older with high risk epMM), high RAMM (younger than 35 years with intermediate or high risk epMM) | HLA Matchmaker | TCMR |
| Sakamoto et al., 2020 | Kidney | HLA Matchmaker, PIRCHE | 100 | Adult | A, B, DR, DQ | DRB/DQB | PIRCHE > 176, epMM ≥ 17 DRB/DBQ | HLA Matchmaker, PIRCHE | dnDSA |
| San Segundo et al., 2022 | Kidney | HLA Matchmaker | 65 | Adult | A, B, C, DRB1, DQA1/B1 | - | >3 (DQB1) | HLA Matchmaker | dnDSA, CXCL10 (urinary) |
| Sapir-Pichadze et al., 2015 | Kidney | HLA Matchmaker | 156 | Adult | A, B, C, DRB1, DQA1/B1 | DR/DQ | >10 (DR), >17 (DQ) | HLA Matchmaker | Graft dysfunction |
| Sapir-Pichadze et al., 2019 | Kidney | HLA Matchmaker | 118,382 | Mixed | A, B, C, DRB1, DQB1 | DRB | - | - | - |
| Senev et al., 2022 | Kidney | HLA Matchmaker | 926 | Adult | A, B, C, DRB1/3/4/5, DQA1/B1, DPA1/B1 | DQA1/B1 | >15 | HLA Matchmaker | - |
| Sharma et al., 2020 | Kidney | HLA Matchmaker | 59 | Pediatric | A, B, C, DRB1/3/4/5, DQA/B | DR/DQ | - | - | - |
| Shin et al., 2022 | Liver | HLA Matchmaker | 172 | Pediatric | DRB1/3/4/5, DQA1/B1 | DQ2/7 | >20 (DR), >22 DQ | HLA Matchmaker | dnDSA |
| Silva et al., 2010 | Kidney | HLA Matchmaker, HLA-EMMA | 62 | Adult | A, B | - | >8 (A), >6 (B), >10 (Class I total) | - | - |
| Singh et al., 2016 | Kidney | HLA Matchmaker | 66 | Adult | A, B, DR, DQ | DQ | - | - | - |
| Snanoudj et al., 2019 | Kidney | HLA Matchmaker | 89 | Adult | A, B, DR, DQ, DP | DQ7 | >27 (Classes I + II combined) | HLA Matchmaker | dnDSA |
| Sullivan et al., 2015 | Heart | HLA Matchmaker | 4,851 | Pediatric | A, B, DR | A, B | Low risk: < 10, Medium risk: 10-20, High risk: >20 (Class I) | HLA Matchmaker | Graft loss |
| Sypek et al., 2020 | Kidney | HLA Matchmaker | 196 | Pediatric | A, B, DRB1/3/4/5, DQA1/B1 | - | >10 (Class I) | HLA Matchmaker | Graft loss |
| Tafulo et al., 2019 | Kidney | HLA Matchmaker | 151 | Adult | A, B, C, DRB1/3/4/5, DQA1/B1 | DR, DQ | DR low risk: <5, DR Medium risk: 5-10, DR High risk: >10, DQ Low risk: <1, DQ Medium risk: 2-5, DQ high risk: >=6 | HLA Matchmaker | ABMR, TCMR |
| Tafulo et al., 2021 | Kidney | HLA Matchmaker | 96 | Adult | A, B, C, DRB1, DQB1 | DRB1, DQB1 | - | - | - |
| Tang et al., 2021 | Kidney | HLA Matchmaker | 366 | Adult | DPB1 | DPB1 | - | - | - |
| Thammanichanond et al., 2018 | Kidney | HLA Matchmaker | 1 | Adult | A, B, DRB1/3/4/5, DQA1/B1, DPA1/B1 | DQA1, B1 | - | - | - |
| Thammanichanond et al., 2020 | Kidney | HLA Matchmaker | 1 | Adult | A, B, C, DRB1/3, DQA1/B1, DPA1/B3 | DPB1*31:01, DPA1*02:02, DPA1*02:02 | - | - | - |
| Tran et al., 2024 | Kidney | HLA Matchmaker | 21 | Adult | DRB1/3/4/5, DQA1/B1 | DQ | *Based on Weibe et al., 2017* | - | - |
| Walton et al., 2016 | Kidney | HLA Matchmaker | 175 | Adult | A, B, DRB1/3/4/5 | DR | >60 (A, B, DRB1/3/4/5), >48 (DQA/B) | HLA Matchmaker | Graft dysfunction |
| Walton et al., 2018 | Lung | HLA Matchmaker | 49 | - | A, B, DRB1/3/4/5, DQA1/B1 | DQA1/B1 | - | - | - |
| Wen et al., 2021 | Kidney | HLA Matchmaker | 771 | - | A, B, C, DRB1, DQA1/B1 | - | Low risk: 27-60, Medium risk: 62-72, High risk: 73-129 | HLA Matchmaker | - |
| Wiebe et al., 2013 | Kidney | HLA Matchmaker | 286 | Mixed | A, B, C, DR, DQ, DP | DQ, DR | >10 (DR), >17 (DQ) | HLA Matchmaker | dnDSA |
| Wiebe et al., 2015 | Kidney | HLA Matchmaker | 195 | Adult | A, B, DR, DQ | DR, DQ | >17 (DQ), >10 (DR) | HLA Matchmaker | ABMR, TCMR, Graft loss |
| Wiebe et al., 2019 | Kidney | HLA Matchmaker | 664 | Pediatric | DRB1/3/4/5, DQA1/B1 | DQ | DR Low risk: 0, DR Medium risk: 1-6, DR High risk: >7; DQ Low risk: 0, DQ Medium risk: 1-8, DQ High risk: >9; DQ Extra-high/most risk >15 | HLA Matchmaker | dnDSA, ABMR, TCMR, Graft loss |
| Wong et al., 2024 | Kidney | HLA Matchmaker | 319 | Adult | A, B, C, DRB1/3/4/5 | DR, DQ | >7 (DR); DQ Low risk: < 9, DQ Medium risk: ≥ 9, < 15, DQ High risk: ≥ 15 | HLA Matchmaker | dnDSA |
| Yanyiam et al., 2024 | Kidney | HLA Matchmaker | 150 | Adult | A, B, DR, DQ | DQ | >9 (A, B); >3 (DR); >2 (DQ) | HLA Matchmaker | dnDSA |
| Zhang J et al., 2020 | Heart | HLA Matchmaker, PIRCHE | 548 | - | A, B, C, DRB1/3/4/5, DQB1 | DQB1 | >21 | PIRCHE | dnDSA |
| Zhang X et al., 2020 | Lung | HLA Matchmaker | 59 | Adult | A, B, C, DR, DQ | DQB1 06:01, C07:02 | - | - | - |
| Zheng et al., 2021 | Kidney | HLA Matchmaker | 124 | Adult | A, B,Cw,DQ,DQ,DP | B | - | - | - |

**Supplementary Table 1.** Brief review of all included articles by graft, HLA matching algorithm, recipient sample size, age cohort, analyzed HLA molecules, most associated HLA molecules with outcomes, reported eplet mismatch thresholds, and adverse outcomes associated with eplet mismatch thresholds.
